# Occlusion-Robust Cattle Pose Estimation for Precision Livestock Monitoring Using Hierarchical Locality Refinement

**DOI:** 10.3390/ani16162575

**Published:** 2026-08-18

**Authors:** Yingchao Wang, Na Li, Dan He, Shan Sun, Xinjian Chu, Zixiang Qin, Feng Xue, Jingjun Yi, Hao Wu, Han-Su Zhang, Fan Zhao

**Affiliations:** 1 Department of Computer Science and Technology, Xinjiang College of Science & Technology, Korla 841000, China; 2 College of Oceanography and Space Informatics, China University of Petroleum (East China), Qingdao 266580, China; 3 Graduate School of Information, Production and Systems, Waseda University, Kitakyushu 808-0135, Japan; 4 State Key Laboratory of Information Engineering in Surveying, Mapping and Remote Sensing, Wuhan University, Wuhan 430079, China; 5 Graduate School of Information Science and Technology, The University of Tokyo, Tokyo 113-8656, Japan; 6 School of Artificial Intelligence and Information Technology, Nanjing University of Chinese Medicine, Nanjing 210023, China; 7 Graduate School of Frontier Sciences, The University of Tokyo, Kashiwa 277-8561, Japan

**Keywords:** cattle pose estimation, keypoint localization, hierarchical locality refinement, ROI refinement, instance-level contrastive regularization, precision livestock farming

## Abstract

Accurate cattle pose estimation is important for precision livestock farming because body landmarks can provide quantitative visual inputs for potential health monitoring, behavior analysis, lameness assessment, and welfare evaluation. This study focuses on keypoint-localization performance and provides a foundation for future task-specific studies of these downstream outcomes. However, real farm images commonly contain inter-cattle occlusion, cluttered backgrounds, small anatomical landmarks, and visually similar animals, which limit the reliability of existing one-stage pose estimators. This study proposes LEC-Pose, a hierarchical locality refinement framework for occlusion-robust cattle pose estimation. Built on YOLOv8-Pose, LEC-Pose first predicts cattle boxes and auxiliary coarse keypoints, then extracts instance-level ROI features to recover local anatomical evidence. A lightweight refinement network directly predicts the final keypoints from heatmaps and offsets on enhanced ROI features. The detected boxes guide the ROI pathway, while the initial keypoints remain auxiliary outputs. During training, an instance-level contrastive loss regularizes a compact global ROI descriptor. During inference, pair construction and contrastive-loss computation are removed, while descriptor fusion remains in the prediction path. Across three runs on CattleEyeView, LEC-Pose reaches 39.47 ± 0.33 mAP@50:95, compared with 33.91 ± 0.28 for YOLOv8-Pose, while running at 111.6 FPS versus 138.2 FPS for YOLOv8-Pose, corresponding to a 19.2% throughput reduction on the evaluated RTX 4090. On NWAFU-Cattle, it obtains 76.08 ± 0.36 and 80.03 ± 0.38 mAP@50:95 under the 50%/50% and 80%/20% protocols, respectively; the former is comparable to FSMC-Pose, whereas the latter is the highest mean among the three repeated principal models. An end-to-end zero-shot evaluation on three external cattle datasets, second-dataset occlusion analysis, detailed module combinations, and descriptor diagnostics further assess the framework within their stated protocols. These results suggest that LEC-Pose can serve as a pose-estimation component for future automated cattle monitoring, and future task-specific studies can connect these keypoints to health, behavior, and welfare outcomes.

## Simple Summary

Accurate cattle pose estimation can support automated monitoring in precision livestock farming, but farm images often contain occlusion, cluttered backgrounds, small body landmarks, and visually similar animals. This study introduces LEC-Pose, which first detects each cattle instance and then refines its keypoints using local region features, heatmaps, offsets, and instance-level representation learning. Experiments on multiple cattle datasets show that the method improves keypoint localization, especially in crowded and occluded scenes, while retaining real-time inference on the evaluated GPU. The method provides a reliable visual basis for future cattle behavior, health, and welfare analysis.

## 1. Introduction

Pose estimation aims to localize semantic anatomical landmarks from images or videos and has become a fundamental task in computer vision. Precision livestock farming combines sensing, computer vision, and data-driven decision support to enable continuous and non-invasive livestock management [1,2]. In human-centered scenarios, accurate keypoint localization has supported a wide range of applications, including motion analysis, action recognition, human–computer interaction, and behavior understanding [3,4,5]. Beyond human analysis, pose estimation is increasingly important for animal behavior understanding and precision livestock farming, where body posture, limb configuration, and motion patterns provide structured visual cues that cannot be fully represented by bounding boxes or image-level labels alone [6,7]. For cattle farming, reliable pose estimation can provide visual cues for potential lameness analysis, feeding and grazing behavior understanding, health monitoring, and welfare assessment in large-scale farms [8]. The present work focuses on pose-estimation accuracy and provides a foundation for future task-specific studies of these downstream outcomes. Compared with manual observation, vision-based pose estimation enables continuous and non-invasive monitoring, making it a promising perception component for automated livestock management systems. Recent visual monitoring studies have also shown that deep learning can support dairy cow behavior recognition and multiview individual cattle behavior analysis in practical barn environments [9,10]. Across other intelligent-monitoring domains, deep models have combined transfer learning with spatial–temporal features, attention-guided generative image modeling, object recognition and multi-object tracking, line-feature visual–inertial localization, and attention-based sequence learning for dynamic signals [11,12,13,14,15,16,17]. Related manufacturing research has modeled machine-tool motion and acceleration and analyzed coated-tool wear mechanisms under demanding cutting conditions, providing complementary examples of structured system and degradation analysis in complex engineering processes [18,19,20]. Autonomous sensing platforms also require navigation and control strategies that remain stable under environmental disturbances and actuator faults [21].

Modern pose estimation methods have developed along several complementary directions. Top–down methods first detect each person or animal instance and then estimate keypoints within the detected region; representative heatmap-based approaches such as SimpleBaseline and HRNet improve localization accuracy through strong convolutional backbones and high-resolution spatial representations [3,4]. Bottom–up methods, such as HigherHRNet, detect all keypoints first and then group them into instances, which improves scalability in crowded scenes but also makes keypoint association more difficult [22]. Another important line of research focuses on coordinate representation and decoding. UDP and DARK reduce systematic bias in heatmap generation and decoding, while SimCC reformulates keypoint localization as coordinate classification to improve precision [23,24,25]. More recently, transformer-based methods such as ViTPose and ViTPose++ have shown strong scalability and transferability for generic body pose estimation, while RTMPose, RTMO, and YOLO-Pose emphasize real-time deployment by improving the accuracy–efficiency trade-off of detection-style or one-stage pose estimators [5,26,27,28,29,30]. The YOLOv8-Pose implementation used in this study was implemented using Ultralytics YOLO version 8.0.196 [31].

Animal pose estimation has also progressed from task-specific supervised models toward more general, transferable, and scalable systems. Early toolkits such as DeepLabCut and SLEAP made markerless animal pose estimation practical by supporting user-defined body parts, efficient annotation workflows, and multi-animal tracking [32,33]. Large-scale benchmarks further expanded the scope of animal pose research. AP-10K introduced a large mammal pose benchmark across diverse animal families and species, APT-36K emphasized both animal pose estimation and tracking in videos, and Animal Kingdom provided large-scale annotations for animal behavior understanding and pose estimation [34,35,36]. Recent studies have moved toward more general animal or mammal pose models. SuperAnimal learns pretrained pose models that can be adapted to many species with reduced manual labeling effort, while KITPose explores instance-level priors and structural dependencies among keypoints for general mammal pose estimation [7,37]. For cattle specifically, CattleEyeView and NWAFU-Cattle provide realistic benchmarks for top-view surveillance videos and side-view multi-cattle images, respectively, enabling systematic evaluation under farm-specific occlusion, scale variation, and background clutter [38,39].

**Figure 1:**
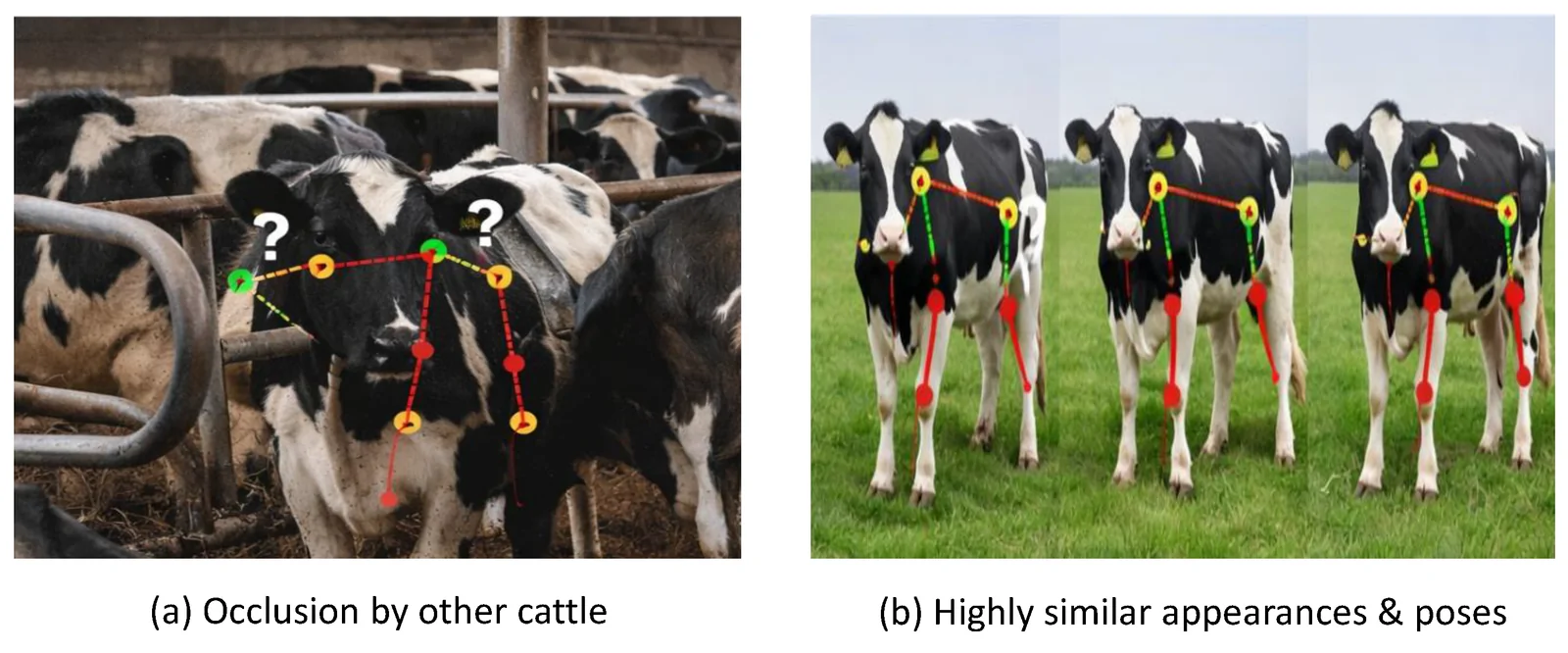
Key challenges in cattle pose estimation. (**a**) Cattle in farm scenes are frequently occluded by nearby animals, railings, and other facilities, making full-body keypoint localization unreliable. (**b**) Different cattle may show similar textures, body shapes, and pose configurations, causing ambiguity in instance association and keypoint localization.

Despite these advances, cattle pose estimation in real farm environments remains challenging because the target scenes differ substantially from common human or generic animal pose benchmarks. Farm images often contain railings, feeding equipment, muddy ground, shadows, illumination changes, and other visual distractors, many of which appear close to small anatomical landmarks such as hooves, knees, ears, and the tail base. Cattle are quadrupeds with elongated bodies and frequent limb self-occlusion, and multiple animals often stand, walk, or feed closely within the same field of view. Within a herd, similar body proportions and repeated coat patterns can make instance boundaries and distal limb landmarks difficult to distinguish, particularly when forelimbs and hindlimbs overlap. As illustrated in Figure 1, nearby cattle and farm facilities can occlude key body parts, while different cattle may exhibit highly similar textures, body shapes, and pose configurations. These factors make cattle pose estimation a joint problem of instance separation, local landmark recovery, and instance-level disambiguation. Similar difficulties have also been observed in cattle behavior recognition and tracking, where inter-cow occlusion, facility obstruction, scale variation, and identity switching remain important bottlenecks for robust barn monitoring [40].

Existing pose estimation paradigms provide useful foundations, yet performance can still degrade when cattle-specific occlusion, crowding, small landmarks, and farm clutter occur simultaneously. Top–down models can achieve strong localization accuracy when instance boxes are reliable, yet the detector can become less reliable under heavy occlusion and the two-stage pipeline can introduce additional computation and error propagation. Bottom–up models are attractive in crowded scenes, but their grouping step can be affected by association errors when neighboring cattle have similar limbs, textures, and body configurations. Transformer-based models improve long-range context modeling and transferability, but they usually require sufficient data and non-trivial computational resources, which can be limiting for practical farm deployment. Real-time one-stage models are more suitable for continuous monitoring because they integrate detection and keypoint prediction in a single forward pass. YOLOv8-Pose is therefore an efficient baseline for cattle pose estimation, but its keypoint prediction is mainly derived from downsampled feature maps and box-relative regression. Under occlusion and inter-instance similarity, fine anatomical cues around legs, hooves, and the tail base can be weakened by feature aggregation or confused with neighboring animals.

These observations motivate a cattle-oriented refinement strategy whose central principle is to recover instance-level local anatomical evidence while strengthening instance-level ROI representations. Instead of treating cattle pose estimation as a single-pass regression task, we formulate it as a locality-enhanced coarse-to-fine process. The coarse stage retains the efficiency of YOLOv8-Pose by detecting cattle instances and predicting auxiliary initial keypoints. The refinement stage then revisits each detected cattle region, extracts fixed-size ROI features with ROIAlign, and enhances them with lightweight local operators [41,42,43,44]. This design narrows the search space from the full image to each cattle instance, reducing interference from nearby animals, farm facilities, and cluttered backgrounds. More importantly, it restores keypoint-sensitive local cues that are often lost in downsampled global features, making the model more responsive to small or partially occluded anatomical landmarks.

**Figure 2:**
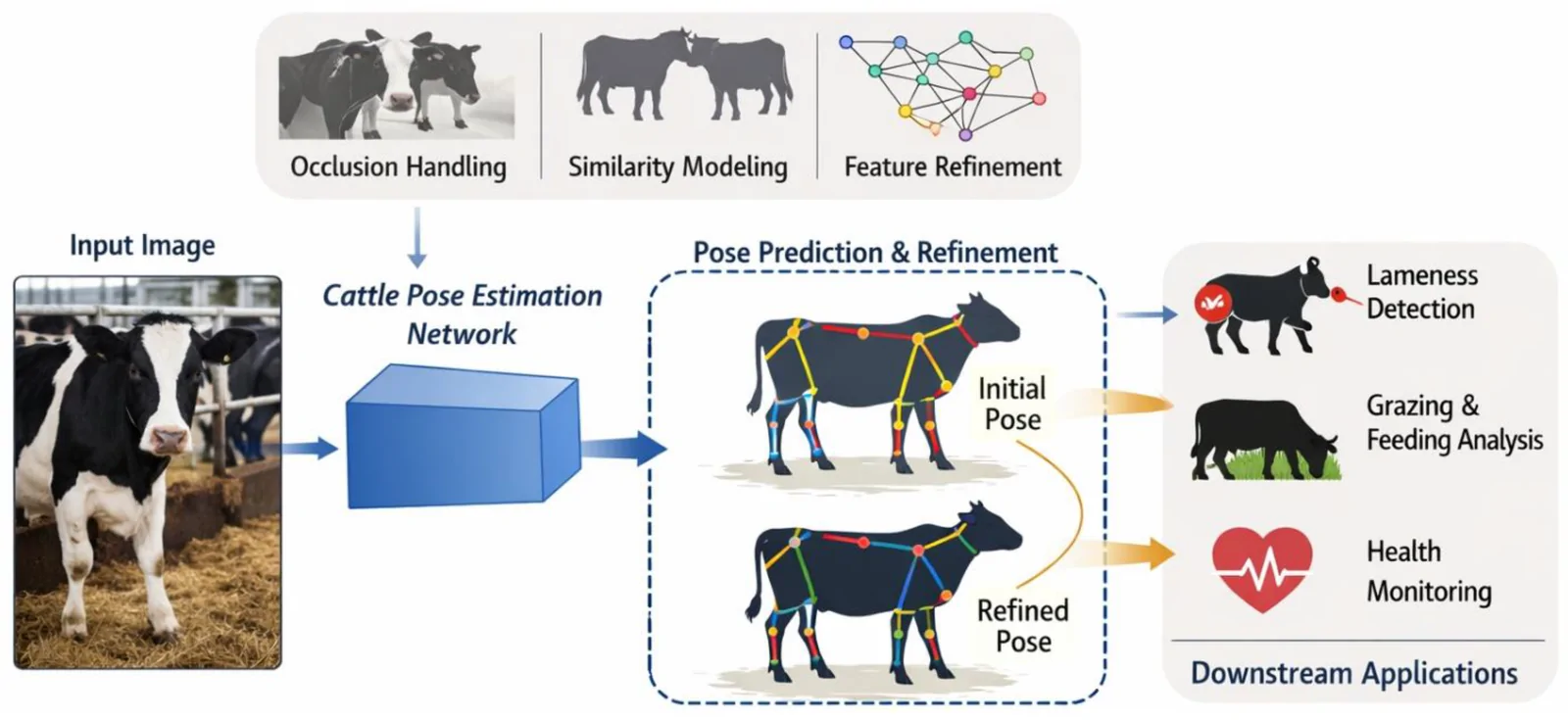
Overview of LEC-Pose for occlusion-robust cattle pose estimation. The model first obtains cattle boxes and auxiliary initial keypoints with a YOLOv8-Pose backbone, then uses the boxes to guide ROI locality enhancement and direct second-stage heatmap-and-offset prediction. The downstream tasks illustrated on the right represent potential applications beyond the pose-estimation experiments reported in this study.

Based on this principle, we propose LEC-Pose, a hierarchical locality refinement framework for occlusion-robust cattle pose estimation. Built upon a YOLOv8-Pose backbone, LEC-Pose introduces three complementary components: part-aware ROI locality enhancement, a second-stage RNet pose head, and instance-level contrastive regularization. The part-aware ROI module determines where fine anatomical evidence should be recovered by extracting and enhancing per-instance local features. RNet directly predicts the final keypoint heatmaps and offset maps from the enhanced ROI features. The detected box defines the ROI, and the coarse keypoints remain auxiliary outputs of the one-stage head. In parallel, the pose embedding branch learns compact global ROI descriptors and optimizes a contrastive objective inspired by discriminative representation learning [45,46]. The objective encourages consistency between selected views of the same cattle instance while separating different instances during training. During inference, contrastive pair construction and contrastive loss computation are disabled, while the lightweight descriptor fusion remains in the prediction path with a small additional cost. The resulting architecture couples efficient one-stage instance localization with targeted ROI evidence recovery, direct second-stage keypoint prediction, and global instance-level context, forming a unified refinement pathway for cattle pose estimation under occlusion, crowding, and visual similarity. The overall workflow is illustrated in Figure 2.

The main contributions of this work are summarized as follows:

- We propose LEC-Pose, a hierarchical locality refinement framework for occlusion-robust cattle pose estimation in real farm environments, where clutter, inter-cattle occlusion, and high visual similarity make keypoint localization unstable.
- By reusing the YOLOv8-Pose multiscale feature pyramid and predicted cattle boxes, LEC-Pose constructs part-aware instance-centered ROI representations and performs direct second-stage heatmap-and-offset prediction within the shared one-stage feature pathway, strengthening keypoint-sensitive evidence for small and occluded landmarks.
- A global ROI descriptor is fused into the refinement pathway and regularized by instance-level contrastive supervision during training, providing complementary instance context for distinguishing overlapping cattle with similar appearances and pose configurations.

## 2. Related Work

### 2.1. Deep Learning-Based Pose Estimation

Deep learning-based pose estimation has been extensively studied in human-centered scenarios and has formed several representative paradigms. Top–down methods first detect each instance and then estimate keypoints within the detected region. Representative models such as SimpleBaseline and HRNet improve localization accuracy through strong convolutional backbones, heatmap regression, and high-resolution spatial representations [3,4]. In contrast, bottom–up methods detect all keypoints in an image and then group them into different instances. OpenPose and HigherHRNet follow this direction and are attractive for crowded scenes, although their performance can be affected by grouping errors when different instances overlap heavily [22,47].

Beyond the top–down and bottom–up paradigms, many studies focus on coordinate representation and keypoint decoding. UDP and DARK analyze quantization bias in heatmap generation and decoding, while SimCC reformulates keypoint localization as coordinate classification to improve localization precision [23,24,25]. Transformer-based models have also shown strong representation capacity for pose estimation. ViTPose and ViTPose++ indicate that plain vision transformers can be effective and scalable for body keypoint detection across different settings [5,26]. Real-time methods such as RTMPose, RTMO, and YOLO-Pose improve the accuracy–efficiency trade-off by integrating detection-style prediction with keypoint estimation [27,28,29]. The YOLOv8-Pose implementation used here follows the Ultralytics release [31]. Recent environmental and agricultural monitoring systems similarly combine image restoration, context-aware real-time detectors, diffusion-enhanced preprocessing, and Mamba-style sequence modeling to improve target recognition under low visibility, scale variation, and complex backgrounds [48,49,50,51,52,53]. Related machine-vision studies have also explored explicit crop–weed segmentation and physics-informed condition monitoring, highlighting the value of foreground separation and domain knowledge in challenging sensing environments [54,55]. Lightweight recognition and anchor-free Mamba/DETR designs further illustrate the importance of efficient feature extraction and multiscale target localization under resource constraints [56,57,58].

Although these methods provide strong foundations for pose estimation, their performance may still be constrained when several cattle-farm challenges co-occur. Top–down methods depend on reliable detection boxes, and their performance can degrade when cattle are heavily occluded or tightly grouped. Bottom–up methods are sensitive to grouping errors when neighboring cattle have similar limbs, textures, and body configurations. Transformer-based models improve global representation, but they often require more training data and computational resources. One-stage methods are efficient for deployment, but their keypoint predictions are usually derived from downsampled feature maps, which weakens fine anatomical evidence around small landmarks. These difficulties can be amplified in multi-cattle scenes with occlusion, background clutter, small keypoints, and high inter-instance similarity.

### 2.2. Animal and Livestock Pose Estimation

Animal pose estimation has become an important tool for behavioral analysis, neuroscience, ecology, and precision livestock farming. Early and widely used toolkits, including DeepLabCut, DeepPoseKit, AniPose, and SLEAP, make markerless animal pose estimation practical by supporting user-defined keypoints, efficient annotation workflows, multi-animal tracking, and multi-view 3D reconstruction [32,33,59,60]. These systems reduce the dependence on physical markers and enable researchers to analyze animal movement from images or videos. However, many of them are designed as flexible toolkits or task-specific pipelines, and their performance depends on annotated data quality, camera setup, and the similarity between training and deployment environments. Recent livestock-oriented work has further extended top–down pose estimation to multi-animal farm scenarios, such as multi-chicken pose estimation based on detection and transfer learning [61].

To improve generalization, recent studies have introduced larger animal pose benchmarks and more transferable models. AP-10K provides a large-scale mammal pose benchmark across diverse animal families and species, while APT-36K extends animal pose estimation to tracking-oriented video settings [34,35]. Animal Kingdom broadens animal behavior understanding by providing annotations for multiple tasks, including pose estimation and action recognition [36]. More recently, SuperAnimal learns pretrained animal pose models that can be adapted to many species with reduced manual labeling effort, and KITPose explores structure-supporting dependencies for general mammal pose estimation [7,37]. These works show that animal pose estimation is moving from single-species and task-specific settings toward more general and transferable systems.

Livestock pose estimation introduces deployment-oriented challenges that are less emphasized in generic animal benchmarks. CattleEyeView provides top-view cattle videos for multi-task cattle monitoring, while NWAFU-Cattle focuses on side-view multi-cattle pose estimation under occlusion and scale variation [38,39]. Recent cattle interaction datasets, such as MOUNT-Cattle, further emphasize mounting behavior, body overlap, and posture deformation [62]. Compared with generic animal datasets, livestock scenes contain stronger background clutter, fixed camera viewpoints, repeated body textures, frequent animal interactions, and stricter efficiency requirements for continuous monitoring.

These studies provide useful datasets, tools, and model designs for animal pose estimation, but robust cattle pose estimation still requires methods that explicitly handle instance overlap, weak local landmark evidence, and pose ambiguity in farm scenes. LEC-Pose follows this direction by combining efficient one-stage prediction with instance-level locality refinement and instance-level representation regularization.

### 2.3. Local Feature Refinement and Attention Mechanisms

Fine-grained keypoint localization requires both global context and local anatomical evidence. Although deep backbones can capture semantic information, repeated downsampling weakens small structures such as distal limbs, hooves, ears, and the tail base. Local feature refinement is therefore widely used to recover spatial details after coarse prediction. ROIAlign, originally introduced in Mask R-CNN, provides accurate instance-level feature pooling by avoiding the misalignment caused by quantized RoI operations [41]. For pose estimation, instance-level pooling narrows the search space from the full image to the target body region, allowing the refinement stage to focus on local geometry, part appearance, and fine keypoint evidence.

Attention mechanisms and geometry-adaptive operators further improve local refinement. Channel and spatial attention modules, such as SE, CBAM, and coordinate attention, reweight feature responses to emphasize informative regions and suppress background clutter [43,44,63]. Deformable convolution improves geometric alignment by learning adaptive sampling offsets, which is useful for articulated bodies and shape deformation [42]. These mechanisms are relevant to cattle pose estimation because local keypoint evidence is frequently weak, partially occluded, or mixed with visually similar body parts from neighboring animals.

LEC-Pose builds on this line of work but differs from generic feature reweighting. Instead of applying attention only to full-image features, it explicitly crops each detected cattle instance, enhances the ROI feature with local operators, and performs heatmap-and-offset prediction within the instance region. LEC-Pose reuses the one-stage feature pyramid and applies box-guided second-stage prediction directly on ROI features, with coarse keypoints retained as auxiliary outputs of the first-stage head. This design links local feature recovery with final keypoint prediction, making the refinement process more targeted for small and occluded cattle landmarks.

### 2.4. Contrastive and Discriminative Representation Learning

Discriminative representation learning organizes feature spaces so that related samples are close while unrelated or confusing samples are separated. Contrastive learning has become a widely used strategy for this purpose. Methods such as MoCo and SimCLR learn visual representations through positive and negative pairs, reducing the dependence on dense manual supervision and improving feature separability [45,46]. Although these methods were originally developed for general visual representation learning, their principle is useful for pose-related tasks where local observations are ambiguous and global configuration provides important disambiguation cues. For animal pose estimation, contrastive representation learning has also been combined with pose-related prior information to improve cross-species keypoint localization, which supports the use of discriminative pose embeddings in livestock pose analysis [64]. Prompt-based contrastive vision–language learning has also been used for anomaly detection in complex infrastructure imagery, providing another example of discriminative supervision for fine-grained visual representations [65].

In cattle pose estimation, discriminative learning can provide additional context because multiple animals may exhibit near-duplicate postures, similar textures, and overlapping body regions. Under occlusion, the model may lose key visual evidence for legs, hooves, or the head. Video data and multi-cattle images provide instance-consistency pairs: adjacent frames or augmented views of the same cattle instance can serve as positives, while other cattle instances in the same batch or scene can serve as negatives. Because these pairs are defined by instance identity or augmentation, the learned representation may contain appearance and identity information in addition to pose geometry.

LEC-Pose introduces a global ROI descriptor branch and a contrastive objective to regularize instance-level ROI representations. The objective is used as representation regularization.

## 3. Method

### 3.1. Framework Overview

We present LEC-Pose, a hierarchical locality refinement framework for cattle pose estimation in real farm environments. LEC-Pose is built upon the YOLOv8-Pose backbone and follows a coarse-to-fine design. The hierarchy refers to progressive processing from image-level cattle detection, to instance-level ROI feature recovery, and finally to second-stage heatmap-and-offset prediction. One-stage pose estimators provide efficient cattle detection and initial keypoint prediction, but their coarse coordinate regression is sensitive to occlusion, background clutter, and visually similar cattle instances. LEC-Pose addresses this limitation by restoring instance-level local evidence before final keypoint prediction and by regularizing instance-level ROI representations during training.

**Figure 3:**
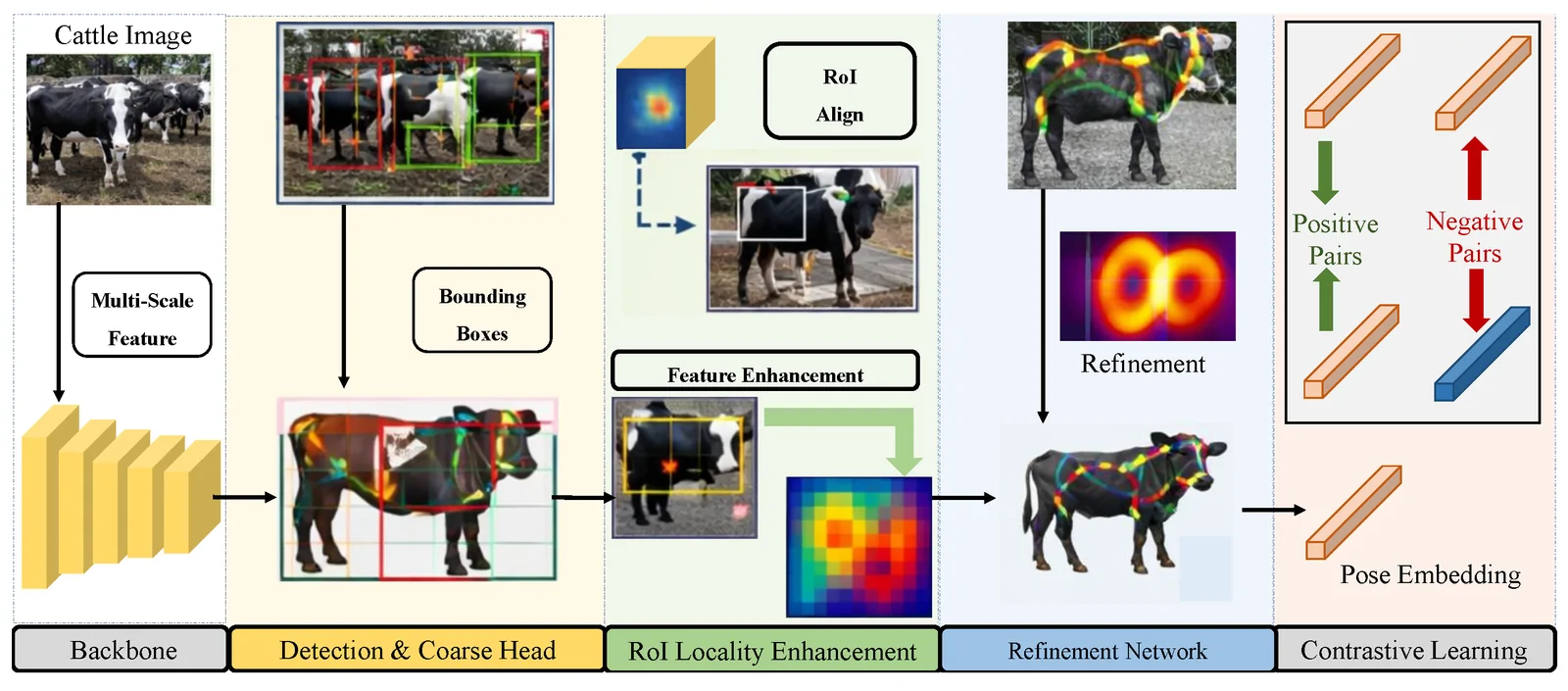
Framework overview of LEC-Pose. Given a cattle image, the YOLOv8-Pose backbone and coarse head first predict cattle boxes and auxiliary initial keypoints. The detected boxes guide ROIAlign to crop instance-level features, which are enhanced by locality refinement operators and fed into RNet for direct heatmap-and-offset prediction. A pose embedding branch extracts compact global ROI descriptors from enhanced ROI features and introduces instance-level contrastive regularization during training. During inference, contrastive pair construction and contrastive loss computation are disabled, and the final keypoints are produced by the detection, ROI locality refinement, descriptor fusion, and RNet prediction pipeline.

Given an input cattle image, LEC-Pose outputs a set of cattle instances, where each instance contains a bounding box, keypoint coordinates, and confidence scores. As shown in Figure 3, the image is first processed by the YOLOv8-Pose backbone and neck to produce multi-scale feature representations. These features are fed into the detection and coarse pose head to predict cattle boxes and auxiliary initial keypoints. This stage preserves the efficiency of one-stage pose estimation and provides coarse instance localization for the following refinement modules.

The coarse head mainly relies on downsampled feature maps and box-relative coordinate regression. Fine anatomical cues around legs, hooves, ears, and the tail base are weakened when cattle overlap with each other or are partially occluded by farm facilities. To restore these cues, LEC-Pose introduces a part-aware ROI locality refinement module. For each detected cattle box, ROIAlign crops an instance-level feature map from the fused pyramid features. Lightweight local operators then enhance the cropped ROI feature, allowing the subsequent refinement stage to focus on keypoint-sensitive regions inside the target cattle instance.

On top of the enhanced ROI features, a lightweight refinement network, termed RNet, directly predicts the final keypoints. The detected box defines the ROI and provides the instance geometry, while the initial keypoints remain auxiliary outputs of the coarse head. RNet predicts keypoint heatmaps and offsets within each ROI. The heatmaps provide dense spatial responses around anatomical landmarks, while the offsets compensate for discretization errors on the ROI grid. This design improves keypoint localization for small or low-contrast landmarks whose OKS scores are sensitive to minor coordinate shifts.

In parallel with keypoint prediction, LEC-Pose introduces a pose embedding branch to provide global instance-level context. The branch extracts compact ROI descriptors from enhanced ROI features and fuses the descriptor into the refinement feature before RNet prediction. During training, the same descriptor is optimized with an instance-level contrastive objective, where selected views of the same cattle instance are pulled closer and embeddings from different instances are pushed apart. During inference, contrastive pair construction and contrastive loss computation are removed; the descriptor fusion remains part of the prediction path.

Overall, LEC-Pose restores locality after efficient one-stage prediction. The YOLOv8-Pose coarse head provides fast instance localization and auxiliary initial keypoints, the ROI locality refinement module recovers instance-level anatomical evidence, RNet directly predicts final keypoints through ROI heatmaps and offsets, and the pose embedding branch regularizes instance-level ROI representations during training. These components form a coherent detection-to-refinement pipeline for cattle pose estimation under occlusion, clutter, visual similarity, and scale variation.

### 3.2. Feature Extraction and Multi-Scale Fusion

Cattle pose estimation requires global context for instance localization and local spatial details for accurate landmark placement. In real farm images, cattle bodies may overlap with each other, while small anatomical parts such as leg joints, hooves, ears, and the tail base often appear in low-contrast or partially occluded regions. A feature extractor that only relies on highly downsampled semantic maps loses fine spatial evidence that is important for keypoint localization. To balance localization accuracy and inference efficiency, LEC-Pose adopts the backbone and neck of YOLOv8-Pose as the multi-scale feature extraction module [31].

Given an input batch $X\in R^{B\times 3\times H\times W}$, the YOLOv8-Pose backbone extracts hierarchical feature maps at multiple spatial resolutions:

(1) $${\left\{F_{\ell }\right\}}_{\ell \in L}=f_{\mathrm{bb}}\left(X\right),\;F_{\ell }\in R^{B\times C_{\ell }\times H_{\ell }\times W_{\ell }},$$

where L denotes the set of feature levels used by the pose head, and the spatial resolution $\left(H_{\ell },W_{\ell }\right)$ decreases as the feature stride increases. Low-level features retain relatively fine spatial details, whereas high-level features provide stronger semantic context for cattle localization.

The neck further fuses these hierarchical features through multi-scale aggregation and produces fused feature maps for detection and coarse pose prediction:

(2) $${\left\{M_{\ell }\right\}}_{\ell \in L}=f_{\mathrm{neck}}\left({\left\{F_{\ell }\right\}}_{\ell \in L}\right),\;M_{\ell }\in R^{B\times {\bar{C}}_{\ell }\times H_{\ell }\times W_{\ell }}.$$

The fused features combine semantically rich high-level information with spatially detailed lower-level information. In LEC-Pose, ${\left\{M_{\ell }\right\}}_{\ell \in L}$ are shared by the detection head, the coarse pose head, and the subsequent ROIAlign operation. This shared design allows the refinement stage to reuse the same multi-scale representations through a single image encoder.

This shared feature design is also important for practical cattle monitoring. Farm systems often process long video streams, so adding a heavy second backbone for refinement would reduce deployment efficiency. By reusing the YOLOv8-Pose backbone and neck, LEC-Pose keeps the early visual encoding lightweight while still providing the information required for hierarchical refinement. The coarse head uses the fused features to obtain cattle boxes and auxiliary initial keypoints, and the ROI locality refinement module reuses the features and boxes to crop instance-level regions and recover local anatomical evidence. Therefore, multi-scale fusion serves as the common feature basis for efficient detection, coarse keypoint regression, and ROI-based second-stage prediction.

### 3.3. Detection and Coarse Pose Head

After multi-scale feature extraction, LEC-Pose adopts a YOLOv8-Pose-style anchor-free head to jointly predict cattle bounding boxes and initial keypoints. This stage is designed to provide fast cattle instance localization and coarse anatomical structure. In farm images, directly regressing all cattle keypoints from full-image features is vulnerable to background clutter, scale variation, and multi-cattle occlusion. By first localizing each cattle instance with a bounding box, the keypoints can be represented in a box-relative coordinate space, which narrows the search region from the full image to the target cattle body and provides spatial anchors for subsequent ROI-based refinement.

Given the fused multi-scale features ${\left\{M_{\ell }\right\}}_{\ell \in L}$, the coarse head predicts a set of candidate cattle detections:

(3) $$C={\left\{\left({\hat{b}}_{m},{\hat{\alpha }}_{m},{\hat{U}}_{m}^{0},{\hat{q}}_{m}^{0}\right)\right\}}_{m=1}^{M}=f_{\mathrm{head}}\left({\left\{M_{\ell }\right\}}_{\ell \in L}\right),$$

where ${\hat{b}}_{m}=\left({\hat{c}}_{m}^{x},{\hat{c}}_{m}^{y},{\hat{w}}_{m},{\hat{h}}_{m}\right)$ denotes the predicted bounding box, ${\hat{\alpha }}_{m}\in \left[0,1\right]$ denotes the cattle instance confidence, ${\hat{U}}_{m}^{0}={\left\{\left({\hat{u}}_{m,i}^{0},{\hat{v}}_{m,i}^{0}\right)\right\}}_{i=1}^{K}$ denotes the coarse keypoint locations in the normalized box coordinate space, and ${\hat{q}}_{m}^{0}={\left\{{\hat{q}}_{m,i}^{0}\right\}}_{i=1}^{K}$ denotes the corresponding keypoint confidences. Here, *M* denotes the number of candidate predictions before post-processing, and *K* is the number of annotated keypoints.

During inference, non-maximum suppression is applied to remove duplicated detections and retain *N* cattle instances:

(4) $$D^{0}={\left\{\left(b_{n},\alpha_{n},U_{n}^{0},q_{n}^{0}\right)\right\}}_{n=1}^{N}=\mathrm{NMS}\left(C\right).$$

For each retained instance, the coarse keypoints are mapped from the normalized box coordinate space to the original image coordinate space. Let $b_{n}=\left(c_{n}^{x},c_{n}^{y},w_{n},h_{n}\right)$, and let $\left(x_{n}^{1},y_{n}^{1}\right)=\left(c_{n}^{x}-w_{n}/2,c_{n}^{y}-h_{n}/2\right)$ be the top-left corner of the box. The image-level coarse coordinate of the *i*-th keypoint is computed as

(5) $$\left(x_{n,i}^{0},y_{n,i}^{0}\right)=\Pi_{b_{n}}\left(u_{n,i}^{0},v_{n,i}^{0}\right)=\left(x_{n}^{1}+w_{n}u_{n,i}^{0},\;y_{n}^{1}+h_{n}v_{n,i}^{0}\right),$$

where $\Pi_{b_{n}}\left(\cdot \right)$ denotes the box-to-image coordinate transform. The resulting coarse pose is denoted as $Y_{n}^{0}={\left\{\left(x_{n,i}^{0},y_{n,i}^{0}\right)\right\}}_{i=1}^{K}$.

The coarse pose $Y_{n}^{0}$ serves as an auxiliary output in LEC-Pose. The predicted box $b_{n}$ guides the subsequent ROI locality refinement and RNet prediction, while $Y_{n}^{0}$ remains the auxiliary output of the one-stage head. Since the coarse head is optimized for efficient global prediction and still relies on downsampled feature maps, its keypoints can be inaccurate for small or occluded landmarks such as hooves, leg joints, ears, and the tail base. The final keypoints are therefore generated by the ROI heatmap-and-offset head described in the following sections.

This detection-and-coarse-pose design provides an efficiency-oriented starting point for the hierarchical refinement pipeline. The anchor-free head rapidly localizes cattle instances and predicts auxiliary initial keypoints, while the box-relative representation reduces the influence of scale variation across animals. The part-aware ROI locality refinement module introduced next complements this global stage by recovering finer local evidence under occlusion, clutter, and inter-cattle overlap.

### 3.4. Part-Aware ROI Locality Refinement

**Figure 4:**
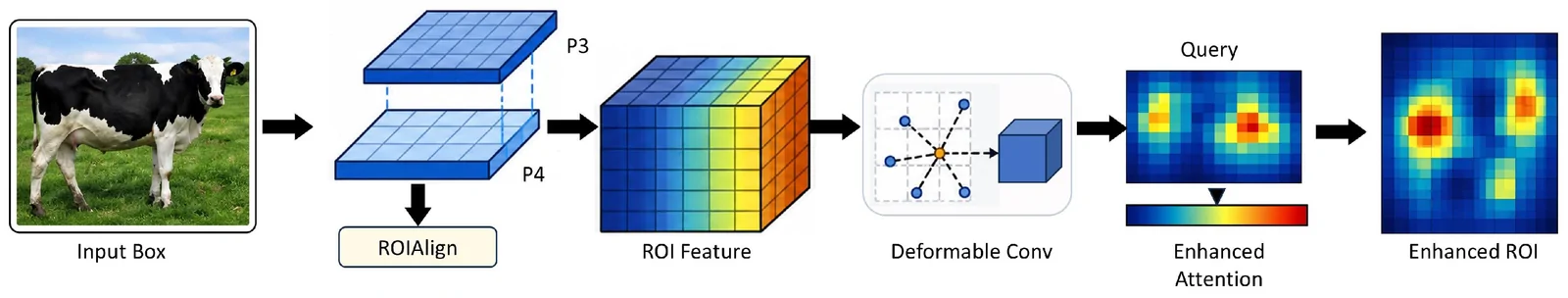
Part-aware ROI locality refinement in LEC-Pose. Given a detected cattle box, ROIAlign crops an instance-level feature map from fused multi-scale features. The ROI feature is processed by deformable convolution to adapt sampling locations to cattle part geometry, and a lightweight attention gate reweights keypoint-related responses. The refined ROI feature is shared by RNet and the pose embedding branch.

Coarse keypoint regression is unreliable for small and partially occluded landmarks. In cattle images, hooves, leg joints, ears, and the tail base occupy limited pixels and are frequently adjacent to fences, shadows, mud, and neighboring body parts. Since the coarse YOLOv8-Pose head predicts keypoints from downsampled pyramid features, local evidence around these landmarks is weakened before coordinate regression. LEC-Pose introduces a part-aware ROI locality refinement module to re-extract instance-level features for each detected cattle box and strengthen local responses before second-stage keypoint prediction. As illustrated in Figure 4, the module consists of three operations: ROIAlign for instance feature extraction, deformable convolution for geometry-adaptive local alignment, and attention reweighting for keypoint-related feature selection.

Given a detected cattle box $b_{n}$, ROIAlign extracts a fixed-size instance feature from the fused multi-scale features:

(6) $$R_{n}=\mathrm{ROIAlign}\left({\left\{M_{\ell }\right\}}_{\ell \in L},b_{n}\right)\in R^{C_{r}\times h_{r}\times w_{r}},$$

where ${\left\{M_{\ell }\right\}}_{\ell \in L}$ denotes the fused feature maps produced by the neck, $C_{r}$ denotes the ROI channel dimension, and $h_{r}$ and $w_{r}$ denote the ROI spatial resolution. For a mini-batch with $N_{r}$ selected cattle instances, the stacked ROI tensor is written as

(7) $$R\in R^{N_{r}\times C_{r}\times h_{r}\times w_{r}}.$$

In our implementation, ROIAlign outputs 32×32 ROI features with 256 channels. This fixed-resolution instance representation removes irrelevant full-image regions and provides a cattle-centered feature source for local keypoint correction.

The extracted ROI feature is processed by deformable convolution to adapt the sampling grid to cattle part geometry. Let K denote the convolutional kernel offsets and let $\Delta p_{n,q}$ denote the learned offset for kernel position *q*. The deformable response at spatial location p is written as

(8) $$R_{n}^{d}\left(p\right)=\sum_{q\in K}W_{q}\;R_{n}\left(p+q+\Delta p_{n,q}\right),$$

where $W_{q}$ is the convolution weight and non-integer sampling locations are evaluated by bilinear interpolation. This operation adjusts feature sampling around bent legs, displaced hooves, and partially visible body parts, producing the geometry-refined ROI feature $R_{n}^{d}$.

The geometry-refined feature is then passed through a lightweight attention gate:

(9) $$A_{n}=\sigma \left(f_{\mathrm{att}}\left(R_{n}^{d}\right)\right),\;R_{n}^{★}=R_{n}^{d}⊙A_{n},$$

where $f_{\mathrm{att}}\left(\cdot \right)$ denotes the attention mapping, σ(·) is the sigmoid activation, and ⊙ denotes element-wise multiplication. The attention map reweights local responses inside the cattle instance. Responses from fences, shadows, mud, or neighboring cattle are suppressed, while responses around anatomical landmarks are amplified. The output $R_{n}^{★}$ is the locality-refined ROI feature.

The part-aware property comes from keypoint-specific refinement supervision. RNet predicts one heatmap and two offset channels for each anatomical landmark on $R_{n}^{★}$. The heatmap and offset losses back-propagate landmark-specific gradients to the ROI feature, driving local activations around different body parts to specialize for different keypoints. Hoof-related responses, leg-joint responses, ear responses, and tail-base responses are optimized through separate keypoint targets using the available landmark annotations. Thus, part awareness is implicit and keypoint-supervision driven.

The refined ROI feature $R_{n}^{★}$ is shared by the refinement network and the pose embedding branch. RNet uses it for direct heatmap-and-offset prediction, while the pose embedding branch uses it to derive a compact global ROI descriptor for feature fusion and contrastive supervision. This shared ROI representation connects second-stage keypoint prediction with instance-level representation regularization: ROIAlign isolates the cattle instance, deformable convolution aligns local part geometry, and attention selects landmark-related evidence. Compared with refinement on full-image feature maps, this module provides a cleaner and more spatially aligned feature source for small, occluded, and visually ambiguous cattle landmarks.

### 3.5. Refinement Network for High-Resolution Keypoints

The coarse pose head predicts box-relative keypoints from downsampled pyramid features. This design keeps the model efficient, but it introduces localization errors on small anatomical landmarks, including hooves, leg joints, ears, and the tail base. These errors become more obvious when keypoints are partially occluded, located in low-contrast regions, or close to body parts from neighboring cattle. RNet performs second-stage keypoint prediction directly from the locality-refined ROI feature $R_{n}^{★}$, with the detected box supplying the instance geometry and the coarse keypoints remaining auxiliary outputs. RNet predicts dense heatmaps and offset maps inside each ROI. The heatmaps describe the spatial response distribution of each landmark, and the offsets provide sub-grid correction for the final coordinate.

Given the refined ROI feature $R_{n}^{★}$, RNet first extracts an intermediate refinement feature:

(10) $$T_{n}=f_{r}\left(R_{n}^{★}\right),$$

where $f_{r}\left(\cdot \right)$ denotes the lightweight convolutional layers in RNet. The heatmap and offset heads then produce two outputs:

(11) $$H_{n}=f_{\mathrm{hm}}\left(T_{n}\right)\in R^{K\times h_{r}\times w_{r}},\;O_{n}=f_{\mathrm{off}}\left(T_{n}\right)\in R^{2K\times h_{r}\times w_{r}}.$$

Here, $H_{n,i}$ denotes the heatmap of the *i*-th keypoint, and $O_{n,i}=\left(O_{n,i}^{x},O_{n,i}^{y}\right)$ denotes its horizontal and vertical offset maps. For a mini-batch with $N_{r}$ selected cattle instances, the stacked outputs are $H\in R^{N_{r}\times K\times h_{r}\times w_{r}}$ and $O\in R^{N_{r}\times 2K\times h_{r}\times w_{r}}$. In our implementation, RNet uses two lightweight convolutional layers with 128 hidden channels before the heatmap and offset heads.

For the *i*-th keypoint, the heatmap logits are normalized over the ROI grid by spatial softmax, producing a probability map $S_{n,i}$. The refined coordinate in ROI space is computed by combining this probability map with the offset maps:

(12) $$\left({\tilde{x}}_{n,i},{\tilde{y}}_{n,i}\right)=\sum_{v=0}^{h_{r}-1}\sum_{u=0}^{w_{r}-1}S_{n,i}\left(v,u\right)\left(u+O_{n,i}^{x}\left(v,u\right),\;v+O_{n,i}^{y}\left(v,u\right)\right).$$

This formulation uses the heatmap to locate the dominant landmark response and uses the offset maps to correct discretization errors on the ROI grid. Compared with a pure soft-argmax on heatmaps, the added offsets preserve sub-grid localization cues and make the refinement more suitable for small cattle landmarks.

The refined ROI-space coordinate is mapped back to the image coordinate system using the detected cattle box. Let $b_{n}=\left(c_{n}^{x},c_{n}^{y},w_{n},h_{n}\right)$, and let $\left(x_{n}^{1},y_{n}^{1}\right)$ be the top-left corner of the box. The final keypoint coordinate is obtained by

(13) $$x_{n,i}=x_{n}^{1}+\frac{w_{n}}{w_{r}-1}{\tilde{x}}_{n,i},\;y_{n,i}=y_{n}^{1}+\frac{h_{n}}{h_{r}-1}{\tilde{y}}_{n,i}.$$

The final keypoint confidence is taken from the heatmap response. During training, ground-truth keypoints are projected into the ROI coordinate system to supervise the heatmap and offset heads. The heatmap target is generated around the projected landmark location, and the offset target records the local displacement from the ROI grid location to the projected ground-truth coordinate. Invalid or invisible keypoints are excluded from the refinement loss.

RNet uses the detected box and enhanced ROI feature to generate the final keypoints, while the coarse keypoints remain auxiliary outputs. The detected box provides the instance region, the ROI feature supplies localized anatomical evidence, the heatmap branch generates dense landmark responses, and the offset branch compensates for fine coordinate errors. This second-stage prediction improves localization for low-contrast, occluded, and spatially crowded cattle keypoints while keeping the additional head lightweight.

### 3.6. Pose Embedding and Instance-Level Contrastive Regularization

Cattle in farm scenes often show near-duplicate pose configurations, such as grazing with slight head movement, standing, and slow walking. When several cattle overlap or share similar textures, ROI features from different instances may become close in the representation space, which increases ambiguity for occluded or weakly visible keypoints. To provide global instance-level context, LEC-Pose introduces a pose embedding branch on top of the locality-refined ROI feature $R_{n}^{★}$. This branch extracts a compact descriptor for each detected cattle ROI, fuses the descriptor into the RNet refinement feature, and applies a contrastive objective during training. CattleEyeView provides temporally adjacent frames with cattle identity information, so ROI features from the same cattle instance in adjacent frames are used as positive pairs. NWAFU-Cattle contains independent images without temporal identity tracks, so two augmented views of the same cattle ROI are used as positives. ROI features from different cattle instances in the mini-batch are treated as negatives. Both strategies encourage same-instance consistency while aligning positive-pair construction with the temporal information available in each dataset. The pair construction targets instance consistency and may preserve appearance and identity cues alongside pose geometry.

Given the refined ROI feature $R_{n}^{★}$, the pose embedding is obtained by global average pooling followed by a projection MLP and $\ell_{2}$ normalization:

(14) $$z_{n}=\frac{g_{\theta }\left(\mathrm{GAP}(R_{n}^{★})\right)}{{∥g_{\theta }\left(\mathrm{GAP}(R_{n}^{★})\right)∥}_{2}}\in R^{d},$$

where $g_{\theta }\left(\cdot \right)$ denotes the projection MLP and *d* is the embedding dimension. In our implementation, *d* is set to 128.

The pose descriptor is also fused into the RNet refinement feature to inject global ROI information into second-stage keypoint prediction. Specifically, a projection layer maps $z_{n}$ to the channel dimension of $T_{n}$, and the projected descriptor is broadcast to the ROI spatial size:

(15) $$E_{n}=\mathrm{Broadcast}\left(h_{\theta }\left(z_{n}\right)\right),\;{\bar{T}}_{n}=f_{\mathrm{fuse}}\left([T_{n},E_{n}]\right),$$

where $h_{\theta }\left(\cdot \right)$ is a linear projection, [·,·] denotes channel-wise concatenation, and $f_{\mathrm{fuse}}\left(\cdot \right)$ is a 1×1 convolution for feature fusion. In the PE variant, ${\bar{T}}_{n}$ replaces $T_{n}$ in the heatmap and offset heads of Equation (11). This design allows the PE branch to influence keypoint prediction even when the contrastive objective is disabled.

Let P(n) denote the positive set of anchor *n*, and let N(n) denote its negative set. The contrastive loss is defined as

(16) $$L_{\mathrm{con}}=-\frac{1}{\left|B\right|}\sum_{n\in B}\frac{1}{\left|P(n)\right|}\sum_{p\in P(n)}\log\frac{\exp(z_{n}^{⊤}z_{p}/\tau )}{\exp\left(z_{n}^{⊤}z_{p}/\tau \right)+\sum_{j\in N(n)}\exp\left(z_{n}^{⊤}z_{j}/\tau \right)},$$

where τ is the temperature parameter and B is the set of ROI embeddings in a mini-batch. The contrastive objective pulls positive views of the same cattle instance closer and pushes embeddings from different cattle instances apart. It therefore regularizes instance-level representation consistency, and the descriptor may encode appearance or identity cues in addition to pose geometry.

During inference, contrastive pair construction and contrastive loss computation are disabled. The pose descriptor fusion remains in the prediction path of LEC-Pose, while the contrastive objective only serves as training supervision. Thus, the descriptor contributes to prediction at inference, while contrastive computation remains a training-only regularizer of the learned ROI representation.

The model is optimized with a weighted multi-task objective that combines cattle detection, coarse keypoint prediction, high-resolution refinement, and instance-level ROI regularization:

(17) $$L_{\mathrm{total}}=L_{\det}+\lambda_{\mathrm{pose}}L_{\mathrm{pose}}+\lambda_{\mathrm{ref}}L_{\mathrm{ref}}+\lambda_{\mathrm{con}}L_{\mathrm{con}}.$$

Here, $L_{\det}$ is the YOLOv8-Pose detection loss for cattle box prediction and instance confidence estimation, $L_{\mathrm{pose}}$ supervises the auxiliary coarse keypoints predicted by the anchor-free pose head, $L_{\mathrm{ref}}$ supervises the RNet heatmap-and-offset outputs, and $L_{\mathrm{con}}$ regularizes the instance-level ROI embedding space. The coefficients $\lambda_{\mathrm{pose}}$, $\lambda_{\mathrm{ref}}$, and $\lambda_{\mathrm{con}}$ balance the four training objectives. We set $\lambda_{\mathrm{pose}}=1.0$, $\lambda_{\mathrm{ref}}=1.0$, and $\lambda_{\mathrm{con}}=0.1$ in all experiments.

## 4. Experiments

This section presents the experimental evaluation of LEC-Pose. We first describe the datasets, evaluation protocols, metrics, and implementation settings. We then compare LEC-Pose with representative pose estimation baselines, coordinate representation methods, and feature enhancement variants. Component-wise ablations and qualitative comparisons are provided to analyze the contribution of each proposed module.

### 4.1. Experimental Setup

#### 4.1.1. Datasets

##### CattleEyeView Dataset

CattleEyeView [38] is a top–down cattle monitoring benchmark containing 14 video sequences, 30,703 frames, and 753 cattle identities. Each cattle instance is annotated with 24 body keypoints covering the head, back, limb joints, hooves, and tail base. The dataset contains frequent inter-cattle occlusion, scale variation, muddy ground, shadows, illumination changes, and cluttered farm backgrounds.

We follow the video-level protocol and split the dataset into 75% training videos and 25% testing videos. The training and testing sets contain disjoint video sequences, which prevents adjacent-frame leakage and evaluates generalization across different monitoring clips.

##### NWAFU-Cattle Dataset

NWAFU-Cattle [39] contains 2432 side-view images and 3101 cattle instances captured in multi-cattle scenes. The dataset includes occlusion, scale variation, side-view body overlap, and ambiguous limb configurations. Since no official split is provided, we evaluate all methods under two image-level protocols with a fixed random seed of 2024.

Protocol A uses 1216 images for training and 1216 images for testing. Protocol B uses 1945 images for training and 487 images for testing. The same image lists are used for all compared methods. The split files are stored as protocol_A_train.txt, protocol_A_test.txt, protocol_B_train.txt, and protocol_B_test.txt. These image-level protocols may contain shared cattle identities, acquisition sessions, camera backgrounds, or near-duplicate content across subsets. They characterize image-level generalization, while the source-stratified and near-duplicate-group-disjoint evaluation below provides a stricter split.

#### 4.1.2. Evaluation Metrics

We evaluate cattle pose estimation using Object Keypoint Similarity (OKS), which measures the agreement between predicted keypoints and ground-truth keypoints after normalizing localization errors by object scale and keypoint tolerance. OKS is defined as

(18) $$\mathrm{OKS}=\frac{\sum_{i=1}^{K}\exp\left(-\frac{d_{i}^{2}}{2s^{2}\kappa_{i}^{2}}\right)I\left(v_{i}>0\right)}{\sum_{i=1}^{K}I\left(v_{i}>0\right)},$$

where $d_{i}$ is the Euclidean distance between the predicted and ground-truth locations of keypoint *i*, $s=\sqrt{wh}$ is the square root of the annotated cattle-box area, $\kappa_{i}$ is the tolerance coefficient of keypoint *i*, and $v_{i}$ is the visibility flag. Neither CattleEyeView nor NWAFU-Cattle provides an official per-keypoint OKS tolerance vector. We therefore use a uniform value $\kappa_{i}=0.05$ for every annotated keypoint on both cattle benchmarks. The same coefficient is used for all compared methods, random seeds, visibility groups, and ablation settings. For an external dataset with an official evaluator, its native tolerance settings are retained; otherwise, the same value is applied to the pre-declared common-keypoint subset.

For metric collection, post-NMS predictions with instance confidence at least 0.001 are retained, with a maximum of 100 cattle instances per image. NMS uses an IoU threshold of 0.65. Predictions are sorted in descending confidence order and matched greedily, one-to-one, to the unmatched ground-truth instance with the highest OKS. A prediction is counted as a true positive when the matched OKS is no smaller than threshold *t*; otherwise, it is a false positive. Unmatched ground-truth instances are false negatives.

At each OKS threshold, the confidence-ranked predictions form a precision–recall curve. We use 101-point interpolated average precision:

(19) $${\mathrm{AP}}_{t}=\frac{1}{101}\sum_{r\in \{0,0.01,\ldots ,1\}}\max_{\tilde{r}\geq r}p_{t}\left(\tilde{r}\right).$$

The reported mAP measures are

(20) $$\mathrm{mAP}@50={\mathrm{AP}}_{0.50},\;\mathrm{mAP}@50:95=\frac{1}{10}\sum_{t\in \{0.50,0.55,\ldots ,0.95\}}{\mathrm{AP}}_{t}.$$

Precision and Recall are taken from the operating point that maximizes the F1 score on the same confidence-ranked curve. Thus, Precision and Recall summarize one operating point, whereas mAP@50 and mAP@50:95 summarize the full curves. Besides accuracy metrics, we report FLOPs, parameter count, FPS, and peak memory under the common implementation protocol.

#### 4.1.3. Implementation Details

All experiments are implemented with Python 3.10.13, PyTorch 2.1.2, torchvision 0.16.2, CUDA 12.1, and cuDNN 8.9.7 on one NVIDIA RTX 4090 GPU with 24 GB memory. YOLOv8-Pose models use Ultralytics 8.0.196. HRNet-, UDP-, DARK-, SimCC-, and ViTPose-based experiments use MMPose 1.2.0 with MMCV 2.1.0. The deformable convolution is implemented with the corresponding MMCV operator. Input images are resized to 640×640 with aspect-ratio padding. All models are trained for 200 epochs with a batch size of 16. We use AdamW with an initial learning rate of $1\times 10^{-3}$, weight decay of $5\times 10^{-4}$, and a cosine learning-rate schedule. A linear warm-up is applied during the first 5 epochs.

YOLOv8-Pose is initialized from the public yolov8n-pose.pt checkpoint. HRNet-W32 and ViTPose-S use the public COCO-pretrained checkpoints distributed with MMPose. mtYOLO uses the authors’ public YOLOv8 multi-task implementation, YOLOX-Pose uses the public livestock-benchmark implementation, and FSMC-Pose uses its official MMPose implementation. Task-specific heads are adapted to the keypoint definition and available labels of the current dataset. For top–down baselines, cattle boxes predicted by the same YOLOv8 detector trained on the corresponding split are used during testing. Ground-truth boxes are not used at test time.

Data augmentation includes random horizontal flipping with probability 0.5, random scaling in [0.75,1.25], random rotation within $\pm 20^{\circ }$, random translation within 0.1 of the image size, and color jittering. For horizontal flipping, left–right keypoint indices are swapped according to the annotation definition. No test-time augmentation is used. The three repeated runs use seeds 2024, 3407, and 7813. Dataset split files are held fixed across seeds; the seed changes model initialization, mini-batch order, and stochastic augmentation only.

During training, each annotated cattle instance provides one ROI. Its ground-truth box is independently perturbed by a horizontal and vertical center shift sampled from [−0.05w,0.05w] and [−0.05h,0.05h], and by a scale factor sampled from [0.90,1.10]. The perturbed box is clipped to the image boundary and discarded only when its width or height becomes smaller than four pixels. At most 32 cattle ROIs are retained per image; when more are available, they are sampled uniformly. ROIAlign uses aligned bilinear sampling, an output size of 32×32, and a sampling ratio of 2. ROI coordinates are detached, so the refinement loss does not back-propagate through box coordinates. During evaluation, post-NMS predicted boxes are used. The 0.001 confidence threshold is used for metric computation, whereas 0.25 is used for FPS measurement and visualization.

The locality module uses one 3×3 deformable convolution with 256 input and output channels, stride 1, padding 1, and one deformable group. Its offset-prediction convolution is initialized to zero. The attention gate consists of a 1×1 convolution reducing 256 channels to 64, a SiLU activation, and a second 1×1 convolution restoring 256 channels, followed by a sigmoid gate. RNet contains two 3×3 convolutions with 128 channels, stride 1, and padding 1; each convolution is followed by batch normalization and SiLU. Separate 1×1 convolutions produce the *K* heatmap logits and 2K offset channels. No spatial upsampling is used because all predictions are made directly on the 32×32 ROI grid.

For a valid keypoint projected to ROI coordinate $\left(x_{i}^{★},y_{i}^{★}\right)$, the heatmap target is a normalized two-dimensional Gaussian with standard deviation σ=1.5 grid cells, truncated at a radius of 3σ. Let $G_{n,i}$ denote this target and $S_{n,i}$ the spatial-softmax prediction. The heatmap loss is

(21) $$L_{\mathrm{hm}}=-\frac{1}{N_{v}}\sum_{n,i}\sum_{u,v}G_{n,i}\left(v,u\right)\log\left(S_{n,i}\left(v,u\right)+10^{-8}\right),$$

where $N_{v}$ is the number of valid supervised keypoints. Offset targets are computed for grid cells within a two-cell radius of $\left(x_{i}^{★},y_{i}^{★}\right)$ as $\left(x_{i}^{★}-u,y_{i}^{★}-v\right)$ and are optimized with Smooth-$L_{1}$ loss. The refinement loss is

(22) $$L_{\mathrm{ref}}=L_{\mathrm{hm}}+0.5\;L_{\mathrm{off}}.$$

Keypoints marked invalid are excluded. Occluded keypoints with valid coordinates remain supervised with the same weight as visible keypoints.

The descriptor projection is a two-layer MLP, 256→256→128, with SiLU between the two linear layers and $\ell_{2}$ normalization at the output. On CattleEyeView, positive temporal pairs are taken from the same cattle identity within a frame distance of at most three; all valid same-identity descriptors in that interval are used as positives. On NWAFU-Cattle, two independently augmented views of the same ROI form the positive pair. All descriptors belonging to other cattle instances in the mini-batch are negatives; no memory queue is used. Anchors without a positive sample are omitted. The temperature is τ=0.07.

The total loss weights are set to $\lambda_{\mathrm{pose}}=1.0$, $\lambda_{\mathrm{ref}}=1.0$, and $\lambda_{\mathrm{con}}=0.1$ in all experiments. $L_{\det}$ follows the YOLOv8-Pose detection loss for box prediction and instance confidence estimation. $L_{\mathrm{pose}}$ supervises coarse keypoints, $L_{\mathrm{ref}}$ supervises RNet heatmap-and-offset outputs, and $L_{\mathrm{con}}$ regularizes the instance-level ROI embedding space. The ROI resolution, RNet width, descriptor dimension, temperature, and loss weights were fixed after pilot validation on held-out training data and were then kept unchanged for every final dataset and test protocol. The 32×32 ROI preserves small hoof and joint responses while limiting quadratic spatial cost; the 128-dimensional descriptor remains compact relative to the 256-channel ROI feature; and $\lambda_{\mathrm{con}}=0.1$ keeps the auxiliary contrastive gradient below the two localization losses.

FLOPs are reported in G under the input resolution of 640×640. For LEC-Pose, FLOPs include the YOLOv8-Pose backbone, neck, coarse prediction head, ROIAlign, ROI locality refinement, RNet prediction, and descriptor fusion. Contrastive pair construction and contrastive loss computation are excluded from inference FLOPs because they are used only during training. FPS is measured with batch size 1 after 50 warm-up iterations and averaged over the evaluation images of each protocol. Image loading, visualization, and file writing are excluded. For NWAFU-Cattle, Protocol A and Protocol B use the same model architecture, so efficiency is reported once for each method. Unless otherwise stated, individual ablation tables report one run per setting, while the principal model comparison is additionally summarized over three repeated runs.

### 4.2. Comparison with Representative Pose Estimation Methods

We compare LEC-Pose with four groups of methods. The first group contains HRNet [4] and YOLOv8-Pose [31]. The second group contains UDP [23], DARK [24], ViTPose [5], and SimCC [25]. The third group contains the SE [43], CBAM [44], MI-CSA [66], Coordinate Attention [63], and MS2AP [67] plug-ins applied to YOLOv8-Pose. The fourth group contains three publicly reproducible livestock-domain baselines: mtYOLO, a YOLOv8-based cattle multi-task model [68]; YOLOX-Pose evaluated with the public livestock keypoint-detection benchmark implementation [69]; and FSMC-Pose, a cattle mounting-pose method designed for cluttered scenes and inter-animal overlap [62]. All applicable methods use the same dataset split, input resolution, end-to-end testing policy, and evaluation implementation as LEC-Pose.

For detector-dependent top–down baselines, the same cattle detector supplies the instance boxes, and no ground-truth boxes are used during the main end-to-end tests. FLOPs include both the shared cattle detector and the pose estimator.

#### 4.2.1. Results on CattleEyeView

Table 1 reports the CattleEyeView results. Compared with YOLOv8-Pose, LEC-Pose improves Precision from 63.80 to 71.26, Recall from 34.80 to 66.73, and mAP@50:95 from 33.80 to 39.58. Among the added livestock-domain baselines, FSMC-Pose reaches 39.03 mAP@50:95, mtYOLO reaches 38.27, and YOLOX-Pose (Livestock) reaches 36.52. LEC-Pose remains 0.55 points higher than FSMC-Pose in mAP@50:95.

#### 4.2.2. Results on NWAFU-Cattle

Table 2 reports the results under the 50%/50% and 80%/20% protocols. The domain-specific comparison gives a more nuanced result than the original generic-only table. Under Protocol A, LEC-Pose has the highest Recall and mAP@50, whereas FSMC-Pose is higher by 0.07 Precision points and 0.06 mAP@50:95 points. Under Protocol B, LEC-Pose has the highest Recall and mAP@50:95, while FSMC-Pose is slightly higher in Precision and mAP@50. These small differences are examined further with repeated training runs below.

#### 4.2.3. Repeated-Run Analysis

Table 3 reports mean and sample standard deviation over three independent runs. On CattleEyeView, LEC-Pose improves mAP@50:95 from 33.91 ± 0.28 for YOLOv8-Pose and 38.96 ± 0.24 for FSMC-Pose to 39.47 ± 0.33. Under NWAFU Protocol A, LEC-Pose and FSMC-Pose are close in descriptive terms, with 76.08 ± 0.36 and 76.17 ± 0.36 mAP@50:95, respectively, while LEC-Pose has the higher Recall mean. Under Protocol B, LEC-Pose reaches 80.03 ± 0.38 mAP@50:95 compared with 79.56 ± 0.48 for FSMC-Pose. Accordingly, performance is reported metric by metric, with Protocol A described as comparable.

#### 4.2.4. Source-Stratified and Near-Duplicate-Group-Disjoint Evaluation

The available NWAFU-Cattle metadata provide source-folder information but lack reliable per-image cattle-identity and video-session labels. We therefore define groups from image provenance and near-duplicate content. Images are first separated by their recorded source folder. Within each source, a 64-bit perceptual hash is computed after resizing to 256×256 grayscale images. An undirected edge is created when the Hamming distance between two hashes is no greater than 6, and each connected component is treated as one group. Complete groups are allocated with seed 2024 while approximately preserving source proportions. The resulting split contains 1689 training images, 248 validation images, and 495 test images, with no connected component shared across subsets. The corresponding files are stored as group_disjoint_train.txt, group_disjoint_val.txt, and group_disjoint_test.txt. This protocol is therefore termed Source-Stratified and Near-Duplicate-Group-Disjoint Evaluation.

Table 4 reports the results under the source-stratified and near-duplicate-group-disjoint split. LEC-Pose obtains 93.88 Precision, 89.74 Recall, 95.36 mAP@50, and 72.84 mAP@50:95. FSMC-Pose has the highest Precision at 94.27, while mtYOLO reaches 70.48 mAP@50:95 and LEC-Pose records the highest Recall and both mAP measures. The result shows that the favorable ranking is retained after near-duplicate groups are separated across subsets.

#### 4.2.5. Detailed Computational Efficiency

Table 5 supplements the existing FLOPs and FPS results with parameter count, peak inference memory, and peak training memory under the same 640-by-640 evaluation setting.

LEC-Pose adds 1.58 million parameters over YOLOv8-Pose. Relative to FSMC-Pose, it uses fewer parameters and nominal FLOPs and runs faster on the evaluated GPU. Relative to YOLOv8-Pose, throughput decreases from 138.2 to 111.6 FPS (19.2%), while nominal FLOPs increase from 9.23 to 12.63 G (36.8%). The measured RTX 4090 result establishes the GPU throughput, and edge-device performance requires platform-specific profiling.

**Figure 5:**
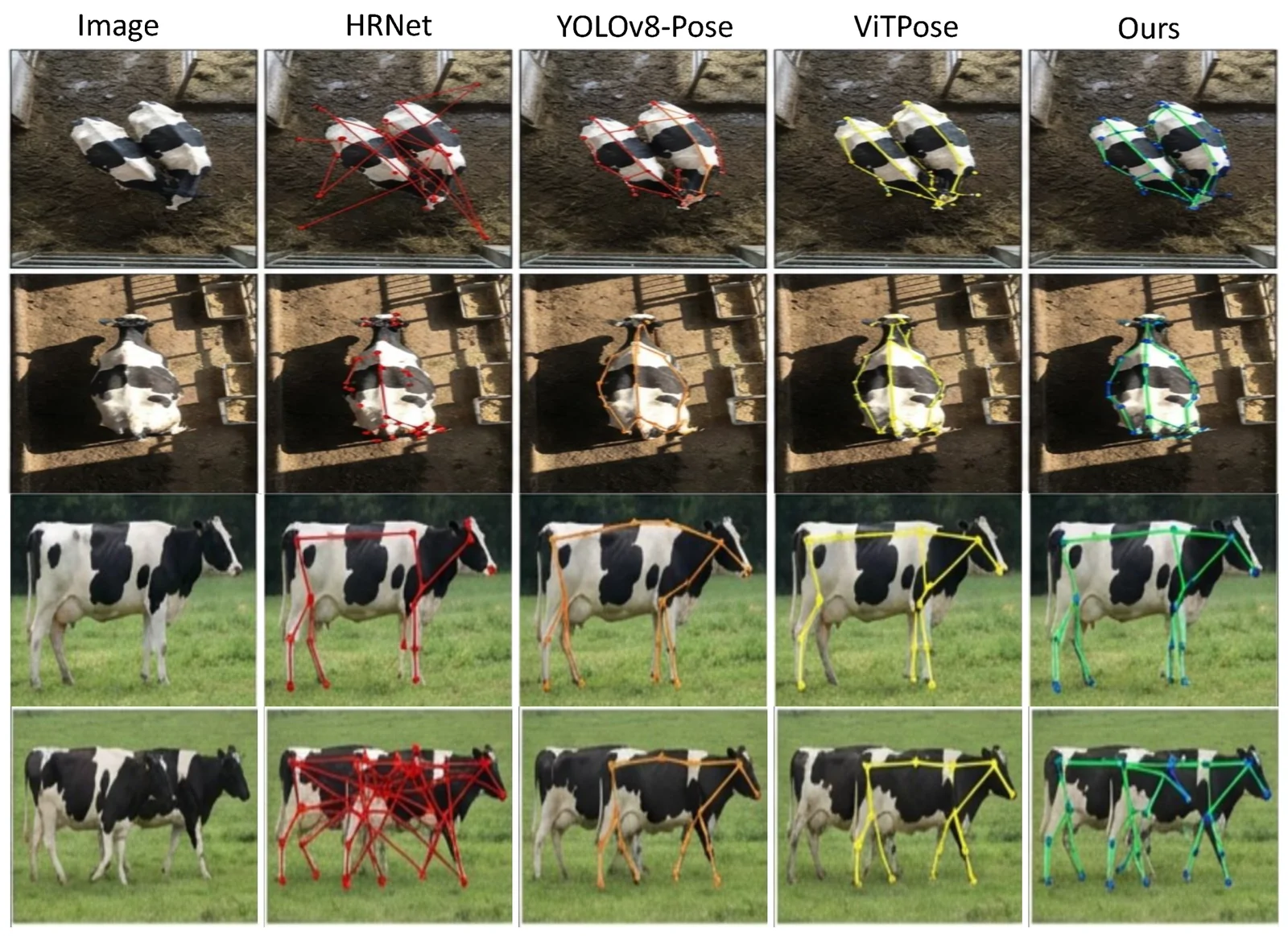
Qualitative comparison between LEC-Pose and representative baselines. The first two rows show CattleEyeView samples with top-view occlusion and crowded cattle instances, and the last two rows show NWAFU-Cattle samples with side-view body overlap and limb ambiguity.

To summarize, the enlarged comparison shows that LEC-Pose is strongest on CattleEyeView and NWAFU Protocol B in mAP@50:95, while Protocol A is comparable to FSMC-Pose under repeated runs. The comparison is reported metric by metric. Representative qualitative comparisons are shown in Figure 5.

### 4.3. Ablation Studies

Table 6 presents a detailed combination study on CattleEyeView to separate the contributions of ROIAlign, the heatmap and offset outputs, standard and deformable convolution, attention, descriptor fusion, and contrastive supervision. All settings use the same data split, optimizer, training schedule, input size, and evaluator.

A1 establishes the standard detector-plus-ROI heatmap baseline and improves mAP@50:95 from 33.80 to 34.42. Adding the offset branch in A2 raises it to 35.14. Relative to standard convolution in A3 (35.58), deformable convolution in A4 reaches 36.16, while the attention-only setting in A5 reaches 35.98. Combining attention with standard convolution in A6 reaches 36.43, and replacing standard convolution with DCN in A7 reaches 36.78. Descriptor fusion adds 1.03 points in A8, and contrastive supervision raises the result to 39.58 in the full A9 model.

#### 4.3.1. Descriptor Representation Analysis

The global ROI descriptor is analyzed by separating identity and pose effects. For each cattle instance, valid ground-truth keypoints are translated by the box center and divided by the box width and height. Pose distance is the mean Euclidean distance over at least eight keypoints valid in both instances. Pairs with distance at most 0.075 are classified as similar-pose pairs, pairs with distance at least 0.185 are classified as different-pose pairs, and intermediate pairs are omitted to avoid ambiguous assignments. Same-identity pairs are required to come from different frames, and at most 10,000 pairs are sampled per category using seed 2024. Table 7 shows that same-identity pairs remain more similar even when their poses differ: the mean cosine similarity for same-identity/different-pose pairs is 0.704, compared with 0.648 for different-identity/similar-pose pairs. The descriptor therefore contains both pose-related and identity- or appearance-related information.

#### 4.3.2. Cross-Backbone Validation with HRNet

To examine cross-backbone compatibility, we attach the same hierarchical locality refinement components to HRNet and evaluate the resulting HRNet-based variants on NWAFU-Cattle. This experiment is used as cross-backbone validation since HRNet serves as the base pose estimator. The same cattle detector used in the main comparison provides instance boxes for HRNet-based variants, and no ground-truth boxes are used during testing. The FLOPs reported in this section include both the shared cattle detector and the HRNet-based pose estimator. PE denotes the pose embedding branch that extracts a compact pose descriptor from the refined ROI feature and fuses it into the refinement feature before keypoint prediction. PE (w/o Con) keeps the pose descriptor fusion but disables the contrastive objective, while PE + Con uses contrastive supervision to regularize the embedding space.

Table 8 reports the HRNet-based validation results under Protocol A. The HRNet baseline achieves 90.43 Precision, 87.19 Recall, and 63.52 mAP@50:95. Adding ROI locality refinement increases the result to 91.58 Precision, 88.03 Recall, and 64.41 mAP@50:95. The gains of 1.15 Precision, 0.84 Recall, and 0.89 mAP@50:95 show that instance-level local feature extraction remains useful even when the base pose estimator already maintains high-resolution representations. ROIAlign guides HRNet features toward cattle body regions and reduces side-view background interference from fences, ground texture, shadows, and neighboring animals.

Adding RNet further improves the result to 92.46 Precision, 89.27 Recall, and 65.83 mAP@50:95. Compared with the ROI-only variant, RNet brings additional gains of 0.88 Precision, 1.24 Recall, and 1.42 mAP@50:95. This result is consistent with HRNet features benefiting from a dedicated ROI-level heatmap-and-offset head. The head directly predicts leg, hoof, and tail-base keypoints within each ROI, improving the reported localization values at stricter OKS thresholds.

With PE but without contrastive supervision, the model reaches 93.21 Precision, 90.12 Recall, and 66.95 mAP@50:95. Compared with the ROI+RNet variant, PE adds 0.75 Precision, 0.85 Recall, and 1.12 mAP@50:95. The descriptor fusion supplies global ROI context to the refinement stage, which can assist prediction of partially visible or locally ambiguous landmarks. After adding contrastive supervision, the full HRNet-based variant obtains 93.87 Precision, 90.76 Recall, and 67.94 mAP@50:95. Compared with PE without contrastive supervision, PE+Con further improves Precision by 0.66, Recall by 0.64, and mAP@50:95 by 0.99. Compared with the HRNet baseline, the full HRNet-based variant improves Precision by 3.44, Recall by 3.57, and mAP@50:95 by 4.42.

Table 9 reports the HRNet-based validation results under Protocol B. The HRNet baseline is stronger under this larger training split, reaching 93.20 Precision, 90.14 Recall, and 65.27 mAP@50:95. ROI locality refinement improves the result to 94.01 Precision, 90.68 Recall, and 66.42 mAP@50:95, with a 1.15 mAP@50:95 gain over the baseline. This result shows that local instance-level feature extraction remains effective when more training images are available. RNet further increases the result to 94.67 Precision, 91.37 Recall, and 67.83 mAP@50:95 through direct keypoint prediction on refined ROI features.

The PE-only variant reaches 95.02 Precision, 92.01 Recall, and 68.94 mAP@50:95, improving over ROI+RNet by 0.35 Precision, 0.64 Recall, and 1.11 mAP@50:95. The full PE+Con variant obtains 95.36 Precision, 92.48 Recall, and 69.83 mAP@50:95, adding another 0.34 Precision, 0.47 Recall, and 0.89 mAP@50:95 over PE-only. Compared with the HRNet baseline, the full HRNet-based variant improves Precision by 2.16, Recall by 2.34, and mAP@50:95 by 4.56. These results show that the proposed modules remain effective when the baseline is stronger and the training set is larger.

Overall, the HRNet-based validation demonstrates compatibility with a second backbone under the evaluated NWAFU-Cattle protocols. Across both protocols, ROI locality refinement improves instance-level feature extraction, RNet provides direct ROI-level keypoint prediction, PE injects global ROI information, and instance-level contrastive supervision is associated with additional single-run gains.

## 5. Discussion

In this section, we further analyze the robustness, supervised target-domain adaptability, and limitations of LEC-Pose beyond the main benchmark comparisons. We first evaluate the behavior of LEC-Pose under keypoint-level occlusion and image-level cattle crowding. We then discuss supervised target-domain evaluation and analyze representative failure cases.

### 5.1. Occlusion Robustness Analysis

Occlusion is a central challenge in real farm cattle pose estimation. To characterize model behavior when keypoints are partially visible or multiple cattle overlap in the same image, we conduct an occlusion-aware analysis on the CattleEyeView test set. We compare three methods: HRNet, YOLOv8-Pose, and LEC-Pose. Results are reported for this dataset and the stated grouping protocol.

We evaluate occlusion robustness from two perspectives. The first perspective groups annotated keypoints by visibility state, including visible, partially occluded, and heavily occluded keypoints. During OKS computation, only keypoints in the corresponding visibility group are used, while keypoints from the other groups are masked out. This protocol evaluates whether a model can localize landmarks when local visual evidence becomes weak or incomplete. The second perspective groups test images by the number of cattle instances, including single-cow images, images with two to three cattle, and images with more than three cattle. This protocol evaluates robustness to inter-cattle interference, where nearby animals introduce overlapping bodies, similar textures, and ambiguous limbs.

#### 5.1.1. Keypoint-Level Occlusion Analysis

For keypoint-level occlusion analysis, each annotated keypoint in the CattleEyeView test set is assigned to one of three visibility groups: visible, partially occluded, and heavily occluded. Two annotators independently reviewed a 64×64 image patch centered on each valid ground-truth keypoint. A keypoint was labeled visible when at least 75% of the local anatomical structure was directly observable, partially occluded when 25–75% remained visible and the location was still directly identifiable, and heavily occluded when less than 25% was visible or the local appearance was absent and the coordinate had to be inferred from surrounding body structure. Disagreements were resolved by joint re-inspection. Keypoints without a valid coordinate were excluded. The same rules were applied to the NWAFU-Cattle Protocol B test set. These categories serve exclusively as evaluation strata, while LEC-Pose outputs keypoint coordinates and confidence scores. The corresponding sample counts are summarized in Table 10.

For each visibility group, we use the same predictions and NMS results as in the main evaluation, but compute OKS only over the keypoints belonging to the target group:

(23) $${\mathrm{OKS}}_{g}=\frac{\sum_{i=1}^{K}I\left(o_{i}=g\right)I\left(v_{i}>0\right)\exp\left(-\frac{d_{i}^{2}}{2s^{2}\kappa_{i}^{2}}\right)}{\sum_{i=1}^{K}I\left(o_{i}=g\right)I\left(v_{i}>0\right)},$$

where *g* denotes the visibility group, $o_{i}$ is the visibility label of keypoint *i*, $v_{i}$ denotes the keypoint validity flag, $d_{i}$ is the Euclidean distance between the predicted and ground-truth keypoint locations, *s* is the cattle instance scale, and $\kappa_{i}$ is the keypoint tolerance coefficient. Instances without valid keypoints in the target group are excluded from that group. Precision, Recall, and mAP@50:95 are computed from the group-specific OKS values.

Table 11 reports the keypoint-level visibility analysis on CattleEyeView. LEC-Pose records the highest values among the three listed methods in all visibility groups. In the visible group, LEC-Pose obtains 76.45 Precision, 72.36 Recall, and 44.67 mAP@50:95. Compared with YOLOv8-Pose, it improves Precision, Recall, and mAP@50:95 by 5.80, 28.64, and 5.76, respectively.

The advantage over YOLOv8-Pose increases as visibility decreases. In the partially occluded group, LEC-Pose reaches 71.18 Precision, 67.84 Recall, and 40.53 mAP@50:95, with gains of 9.00 Precision, 33.32 Recall, and 7.29 mAP@50:95. In the heavily occluded group, LEC-Pose obtains 64.28 Precision, 58.69 Recall, and 35.91 mAP@50:95, improving over YOLOv8-Pose by 13.86 Precision, 33.78 Recall, and 10.18 mAP@50:95. Within this test set, the gain over YOLOv8-Pose increases as local anatomical evidence becomes less complete.

From visible to heavily occluded keypoints, YOLOv8-Pose drops by 20.23 Precision, 18.81 Recall, and 13.18 mAP@50:95, whereas LEC-Pose drops by 12.17 Precision, 13.67 Recall, and 8.76 mAP@50:95. The smaller degradation indicates better preservation of the reported metrics on the CattleEyeView test set. This pattern is consistent with box-guided ROI isolation, local evidence recovery, direct RNet heatmap-and-offset prediction, and global instance-level ROI context.

#### 5.1.2. Image-Level Crowding Analysis

For image-level crowding analysis, each test image is assigned to one of three groups according to the number of annotated cattle instances. Images containing one cattle instance are assigned to the single-cow group, images containing two to three cattle instances are assigned to the 2–3 cows group, and images containing more than three cattle instances are assigned to the more-than-three-cows group. For each group, we use the same predictions and NMS results as in the main evaluation, but compute Precision, Recall, and mAP@50:95 only on the images belonging to that group. This protocol evaluates how each method behaves as inter-cattle interference increases.

Table 12 reports the image-level crowding analysis on CattleEyeView. LEC-Pose records the highest values among the three listed methods in all crowding groups. In the single-cow group, LEC-Pose obtains 74.02 Precision, 70.48 Recall, and 42.35 mAP@50:95, improving over YOLOv8-Pose by 5.65, 30.62, and 5.73, respectively.

For images with two to three cattle, LEC-Pose reaches 71.45 Precision, 67.02 Recall, and 39.83 mAP@50:95, with gains of 7.51 Precision, 31.74 Recall, and 5.91 mAP@50:95 over YOLOv8-Pose. For images with more than three cattle, it reaches 68.36 Precision, 61.15 Recall, and 36.92 mAP@50:95, with corresponding gains of 10.71, 32.68, and 7.18. The larger differences over YOLOv8-Pose indicate better preservation of the reported metrics as inter-cattle interference increases within this dataset.

From single-cow images to images containing more than three cattle, YOLOv8-Pose drops by 10.72 Precision, 11.39 Recall, and 6.88 mAP@50:95, whereas LEC-Pose drops by 5.66 Precision, 9.33 Recall, and 5.43 mAP@50:95. This result is consistent with box-guided instance isolation, local evidence recovery, direct RNet prediction, and global instance-level ROI context.

#### 5.1.3. Occlusion Robustness on NWAFU-Cattle

The second-dataset analysis uses the NWAFU-Cattle Protocol B test set and the same three visibility concepts as the CattleEyeView analysis. Table 13 reports the corresponding sample counts and localization results.

LEC-Pose has the highest mAP@50:95 in all three groups. Its margin over FSMC-Pose increases from 0.45 points for visible keypoints to 3.08 points for partially occluded keypoints and 4.54 points for heavily occluded keypoints. FSMC-Pose is slightly higher in visible-group mAP@50, whereas LEC-Pose has the highest mAP@50 in the partially and heavily occluded groups. The larger margin under stronger occlusion is consistent with the local-evidence recovery motivation.

### 5.2. External-Dataset Evaluation

#### 5.2.1. Supervised Target-Domain Evaluation

We evaluate supervised target-domain performance on seven external animal-pose datasets: three cattle datasets (CowPose, Roboflow-CowPose, and MOUNT-Cattle) and four related quadruped datasets (DairyGoatMVT, Horse-10, BamaPig2D, and StanfordExtra). Table 14 summarizes their viewpoints, species scopes, and scene domains. Every model receives labeled target-domain training data and target-domain validation, so this protocol evaluates adaptation under controlled supervision.

The exact image-level partitions used in the supervised target-domain evaluation are listed in Table 15. Roboflow-CowPose and MOUNT-Cattle retain their released partitions. CowPose, DairyGoatMVT, Horse-10, BamaPig2D, and StanfordExtra use fixed image-level 70%/10%/20% partitions generated with seed 2024. The counts refer to valid images retained after annotation checking.

For every target dataset, all annotations and generated crops from the same image remain in one subset. From the corresponding training partition, 1000 valid annotated instances are sampled without replacement. For releases organized into multiple source folders, the allocation is proportional to the number of valid training instances in each source. A multi-instance image may contribute more than one training instance, but all instances from that image remain in the same subset. For datasets without animal-identity annotations, the partitioning unit is the image. The fixed image and instance lists are shared across all compared methods, and the 1000-sample setting is used as a controlled target-domain annotation budget.

The 1000 original samples are expanded to 3000 training samples through keypoint-preserving augmentation. The augmented set consists of the original samples and 2000 generated samples. The augmentation operations include random horizontal flipping with left-right keypoint remapping, random scaling in [0.75,1.25], random rotation within $\pm 20^{\circ }$, random translation within 0.1 of the image size, random cropping with aspect-ratio padding, color jittering, Gaussian blur, random erasing, and synthetic line occlusion. The synthetic line occlusion inserts thin fence-like strips across the image or ROI to simulate railings and farm facilities. Bounding boxes and keypoint coordinates are transformed together with the image. Samples whose transformed boxes become invalid or whose valid keypoints fall outside the image are discarded and regenerated.

All compared methods are trained on the same target-domain training set for each dataset. HRNet, YOLOv8-Pose, and LEC-Pose use the same training split, validation split, input resolution, and augmentation pipeline. Task-specific prediction heads are replaced according to the keypoint definition of each target dataset. For datasets whose keypoint definitions differ from cattle benchmarks, evaluation is performed using each target dataset’s native keypoint definition and evaluation protocol. Input images are resized to 640×640 with aspect-ratio padding. Each model is trained for 200 epochs with a batch size of 16 using AdamW, an initial learning rate of $1\times 10^{-3}$, weight decay of $5\times 10^{-4}$, and a cosine learning-rate schedule with 5 warm-up epochs. The checkpoint with the highest validation mAP@50:95 is used for final testing. During evaluation, predictions are ranked by instance confidence after NMS, and mAP@50:95 is computed under the target dataset protocol. Related few-shot learning research has investigated layer-adaptive optimization to improve learning efficiency when labeled target data are limited [76]. This protocol measures supervised target-domain adaptation using labeled target data and target-validation model selection. Results are reported within each dataset because keypoint definitions and evaluation protocols differ across datasets.

Table 16 reports the external-dataset results. With 1000 labeled target samples, 200 training epochs, and target-validation checkpoint selection, LEC-Pose records the highest single-run value among the three named methods on each dataset. These results quantify supervised adaptability under the specified target-domain protocols. Zero-shot transfer is evaluated separately in the following subsection.

#### 5.2.2. Zero-Shot Cross-Dataset Evaluation

For zero-shot cross-dataset testing, each model is trained on the NWAFU-Cattle source split and selected using source-domain validation. Target datasets contribute only the final test images and the predeclared keypoint mappings. Each source-trained method is applied end to end with its native inference pipeline. The common 12-keypoint subset for CowPose and Roboflow-CowPose consists of the left and right shoulder, fore-knee, fore-hoof, hip, hind-knee, and hind-hoof landmarks. MOUNT-Cattle uses the same 12 landmarks together with the nose and tail base, giving 14 common keypoints. Left and right are defined from the animal’s anatomical perspective. Target landmarks outside the declared subset are ignored during scoring, and the correspondence is fixed before any target-domain result is inspected. The corresponding zero-shot results are reported in Table 17.

LEC-Pose obtains the highest mAP@50:95 on all three targets: 31.76 on CowPose, 28.43 on Roboflow-CowPose, and 27.08 on MOUNT-Cattle. The gains over mtYOLO are 2.68, 2.47, and 1.89 points, respectively. On MOUNT-Cattle, mtYOLO has the higher Precision and mAP@50, whereas LEC-Pose has higher Recall and mAP@50:95.

### 5.3. Failure Case Analysis, Limitations, and Future Directions

**Figure 6:**
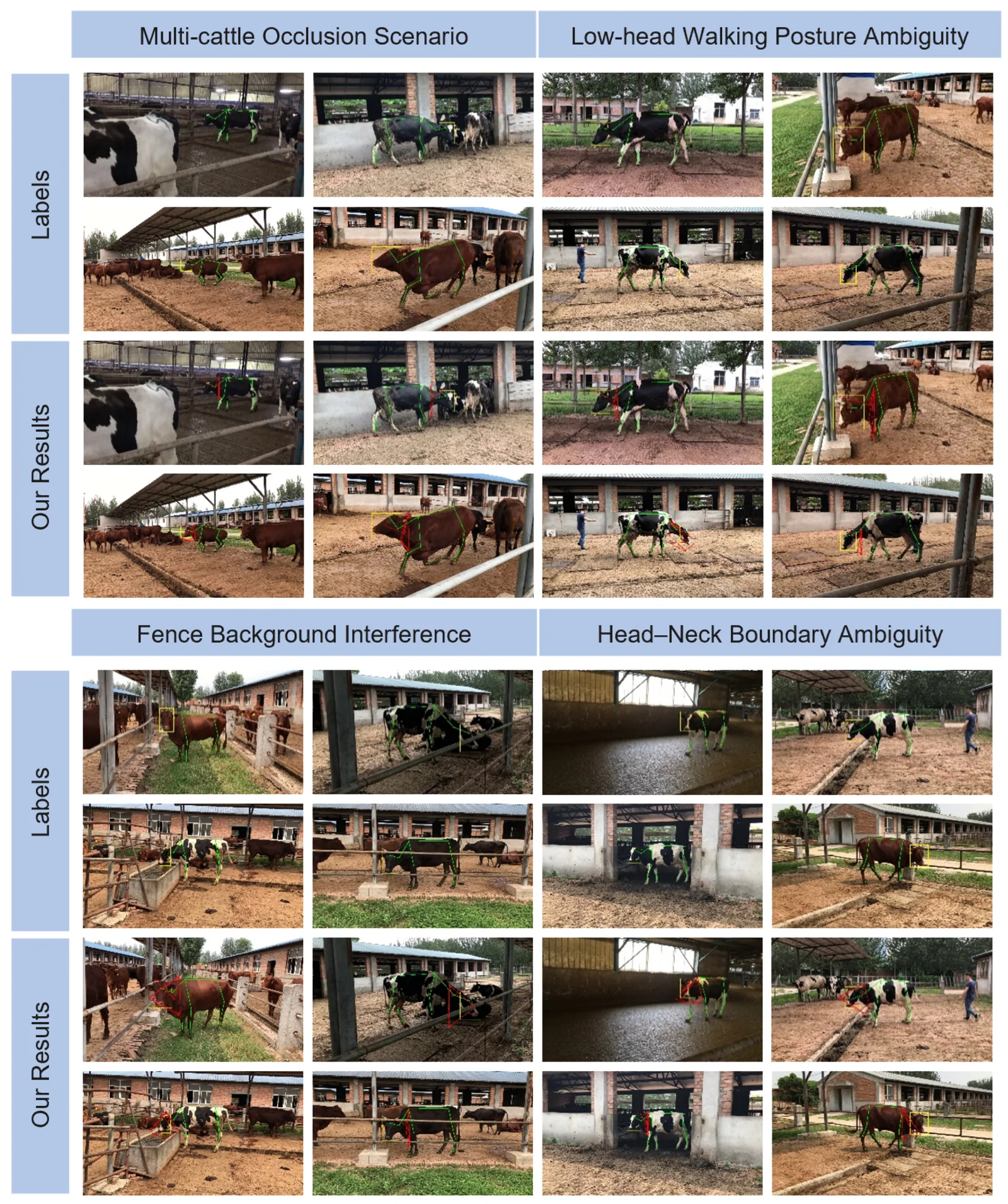
Failure case analysis of LEC-Pose. The figure compares ground-truth labels and LEC-Pose predictions under four challenging scenarios: multi-cattle occlusion, low-head walking posture ambiguity, fence background interference, and head–neck boundary ambiguity.

Although LEC-Pose improves cattle pose estimation under occlusion, crowding, and visual similarity, several challenging cases remain unresolved. Figure 6 summarizes representative failure cases by comparing ground-truth labels with the predictions of LEC-Pose. These cases reveal four major limitations of the current framework: severe multi-cattle occlusion, low-head walking posture ambiguity, fence background interference, and head–neck boundary ambiguity. They indicate that hierarchical locality refinement can reduce many common errors, but extreme missing evidence and structural ambiguity still require stronger temporal, anatomical, and foreground-background reasoning.

The first limitation is severe multi-cattle occlusion. When one cattle instance is largely blocked by another animal, the detected ROI still contains overlapping body regions from multiple cattle. In this case, ROIAlign can isolate the target box but cannot reconstruct missing body parts, and RNet receives incomplete or mixed local evidence. As a result, the model may miss occluded legs or assign hoof and limb keypoints to the wrong animal. Future work can introduce occlusion-aware keypoint visibility modeling, where the network predicts keypoint visibility together with heatmaps and offsets so that invisible or heavily occluded landmarks can be supervised with different confidence and loss weights. Another direction is temporal feature propagation. Since farm videos contain adjacent frames of the same cattle, visible keypoints from neighboring frames can be propagated to the current occluded frame through tracking, temporal attention, or pose memory, reducing errors caused by short-term body overlap.

The second limitation is low-head walking or grazing-like posture ambiguity. In these cases, the head, neck, forelimbs, and shoulder region form compact visual patterns, and the head direction becomes difficult to distinguish from the body contour. LEC-Pose may produce locally plausible predictions that are anatomically inconsistent, especially when the head is close to the ground or partially hidden by the front legs. Future work should incorporate stronger anatomical pose priors. One solution is to learn a cattle-specific skeletal prior that constrains the relative geometry among the head, neck, shoulder, and forelimbs. Another solution is to build a pose-prototype memory, where common cattle postures such as standing, walking, grazing, and lying are represented as learnable prototypes. During refinement, the predicted pose can be adjusted toward a compatible prototype while preserving local image evidence, improving stability for ambiguous low-head postures.

The third limitation is fence background interference. Railings, bars, and farm facilities often have elongated shapes and high contrast, which resemble cattle limbs or body boundaries. When these structures cross the cattle body, local feature responses around legs, hooves, and the neck can be corrupted. The current attention module reweights ROI features, but it does not explicitly separate foreground cattle parts from line-like background objects. Future work should add foreground-aware or boundary-aware supervision. A concrete direction is an auxiliary cattle-foreground or part-segmentation branch that suppresses fence-like structures before keypoint prediction. A boundary-aware loss can also be introduced to separate thin limbs from thin background lines. In addition, synthetic occlusion augmentation with randomly inserted fence-like patterns can increase training diversity and reduce the tendency to treat railings as anatomical landmarks.

The fourth limitation is head–neck boundary ambiguity. In side-view images or low-illumination scenes, the transition between the head, neck, and shoulder can be visually smooth, especially for cattle with similar body textures. Predicted head or neck keypoints may drift along the body contour when the local appearance boundary is weak. Future work should combine keypoint estimation with part-level structural reasoning. A hierarchical representation can first estimate coarse body parts, such as head, torso, and limbs, and then localize fine keypoints within each part. This would give RNet a stronger anatomical reference for separating adjacent landmarks. Multi-scale boundary features can also be added to the ROI branch so that the model learns sharper transitions between neighboring anatomical regions.

Beyond these visual failure modes, LEC-Pose still depends on accurate cattle detection and performs inference mainly from single images. If the detector produces an incomplete box, merges two nearby cattle into one box, or misses a heavily occluded animal, the ROI refinement stage receives an incorrect instance region. Future work can improve the coupling between detection and pose estimation through pose-guided box refinement or joint instance association. Moreover, the current inference pipeline does not explicitly exploit motion continuity, gait periodicity, or cross-frame identity consistency. A video-level extension with temporal ROI aggregation, tracking-guided memory, or recurrent pose refinement could stabilize predictions under temporary occlusion and reduce frame-to-frame jitter in downstream behavior analysis. For multi-farm deployment, communication-efficient and privacy-preserving federated learning could support collaborative model updates while reducing the need to centralize raw farm imagery [77,78].

## 6. Conclusions

In this work, we presented LEC-Pose, a hierarchical locality refinement framework for occlusion-robust cattle pose estimation in real farm environments. The framework combines shared one-stage features, box-guided ROI evidence recovery, direct second-stage heatmap-and-offset prediction, and training-time instance-level ROI regularization. The initial keypoints are auxiliary outputs.

The revised comparison protocol includes three publicly reproducible livestock-domain baselines, together with three-seed repeats for the principal models. LEC-Pose reaches 39.47 ± 0.33 mAP@50:95 on CattleEyeView and 80.03 ± 0.38 under NWAFU-Cattle Protocol B. Under Protocol A, its 76.08 ± 0.36 result is comparable to FSMC-Pose at 76.17 ± 0.36, while LEC-Pose retains the higher Recall mean. Under the source-stratified and near-duplicate-group-disjoint NWAFU-Cattle split, LEC-Pose records 72.84 mAP@50:95 and the highest Recall and mAP values. Detailed combinations show complementary gains from ROI heatmap prediction, offsets, deformable convolution, attention, descriptor fusion, and contrastive supervision.

The second-dataset occlusion analysis shows that the margin over FSMC-Pose increases with occlusion severity, and the end-to-end zero-shot test gives the highest mAP@50:95 for LEC-Pose on CowPose, Roboflow-CowPose, and MOUNT-Cattle. Descriptor diagnostics indicate that the global ROI representation contains both pose geometry and instance-specific appearance information, supporting its interpretation as an instance-level descriptor.

More broadly, reliable cattle pose estimates provide a structured visual representation for future continuous livestock-monitoring systems. Future task-specific studies can translate these estimates into lameness, behavior, health, and welfare decisions. Severe multi-cattle occlusion, fence-like background structures, weak head–neck boundaries, detector errors, and the lack of temporal reasoning remain important limitations. Future work will investigate visibility-aware prediction, temporal feature propagation, anatomical priors, foreground–background separation, detector–pose interaction, and video-level refinement. 

## Figures and Tables

**Table 1:** Comparison with representative methods on CattleEyeView.

Method	Precision (%)	Recall (%)	mAP@50 (%)	mAP@50:95 (%)	FLOPs (G)	FPS
HRNet [4]	45.90	61.50	28.73	12.20	25.2	71
YOLOv8-Pose [31]	63.80	34.80	56.43	33.80	9.2	138
SE [43]	64.93	46.27	57.18	34.46	9.3	137
CBAM [44]	65.81	48.15	58.64	35.52	9.6	127
MI-CSA [66]	62.46	44.84	55.91	33.48	9.7	122
CA [63]	63.95	45.11	57.49	34.73	9.4	135
MS2AP [67]	64.19	48.86	59.87	36.27	9.8	124
UDP [23]	68.92	54.21	63.04	37.20	42.1	46
DARK [24]	69.20	54.98	63.21	37.35	42.1	49
ViTPose [5]	69.05	56.81	63.68	37.47	26.3	67
SimCC [25]	69.24	58.10	64.11	37.55	14.7	105
mtYOLO [68]	68.47	61.92	65.83	38.27	18.54	84.6
YOLOX-Pose (Livestock) [69]	66.13	56.44	62.74	36.52	12.18	119.7
FSMC-Pose [62]	70.08	64.87	67.41	39.03	15.68	96.8
LEC-Pose	71.26	66.73	68.17	39.58	12.60	111.6

**Table 2:** Comparison with representative methods on NWAFU-Cattle. Bold numerical values indicate the best performance for each accuracy metric under the corresponding protocol.

	Protocol A: 50%/50%				Protocol B: 80%/20%				Efficiency	
Method	Precision	Recall	mAP@50	mAP@50:95	Precision	Recall	mAP@50	mAP@50:95	FLOPs (G)	FPS
HRNet [4]	90.43	87.19	93.74	63.52	93.20	90.14	95.21	65.27	25.2	88
YOLOv8-Pose [31]	95.78	72.24	97.31	68.86	97.21	74.38	98.81	72.45	9.2	166
SE [43]	92.16	81.02	97.82	70.59	96.87	83.97	99.03	73.79	9.3	168
CBAM [44]	96.45	83.20	98.06	69.82	95.35	84.26	98.88	74.91	9.6	162
MI-CSA [66]	95.57	83.04	98.21	71.25	97.12	85.18	99.17	75.05	9.7	158
CA [63]	93.72	81.55	97.95	71.91	95.64	85.85	98.72	73.07	9.4	166
MS2AP [67]	94.81	82.68	96.88	67.53	96.23	86.31	98.69	73.30	9.8	155
UDP [23]	96.65	89.49	98.64	73.41	97.15	90.22	99.28	77.40	42.1	76
DARK [24]	95.84	88.68	98.76	74.62	96.27	90.38	99.22	76.65	42.1	75
ViTPose [5]	96.71	88.62	98.53	72.55	97.22	89.35	99.25	76.70	26.3	86
SimCC [25]	95.86	87.74	98.69	73.80	95.71	90.45	99.31	76.80	14.7	124
mtYOLO [68]	97.06	90.74	98.93	75.12	97.93	93.14	99.21	79.04	18.54	105.9
YOLOX-Pose (Livestock) [69]	95.41	87.83	98.11	72.88	96.72	90.32	98.49	76.91	12.18	143.2
FSMC-Pose [62]	**98.34**	92.61	99.08	**76.31**	**98.51**	94.88	**99.47**	79.63	15.68	117.6
**LEC-Pose**	98.27	**93.53**	**99.16**	76.25	98.38	**95.97**	99.43	**80.12**	12.60	132.7

**Table 3:** Three-seed results for the principal models. Values are mean ± sample standard deviation over seeds 2024, 3407, and 7813. Bold numerical values indicate the best performance for each accuracy metric under the corresponding protocol.

Dataset/Protocol	Method	Precision (%)	Recall (%)	mAP@50 (%)	mAP@50:95 (%)
CattleEyeView	YOLOv8-Pose	63.74 ± 0.35	35.18 ± 0.60	56.52 ± 0.39	33.91 ± 0.28
CattleEyeView	FSMC-Pose	70.02 ± 0.46	64.71 ± 0.58	67.34 ± 0.37	38.96 ± 0.24
CattleEyeView	LEC-Pose	**71.11 ± 0.46**	**66.49 ± 0.54**	**68.05 ± 0.36**	**39.47 ± 0.33**
NWAFU-Cattle Protocol A	YOLOv8-Pose	95.84 ± 0.17	72.61 ± 0.59	97.42 ± 0.13	68.74 ± 0.37
NWAFU-Cattle Protocol A	FSMC-Pose	**98.22 ± 0.13**	92.47 ± 0.42	99.02 ± 0.06	**76.17 ± 0.36**
NWAFU-Cattle Protocol A	LEC-Pose	98.19 ± 0.16	**93.31 ± 0.39**	**99.14 ± 0.05**	76.08 ± 0.36
NWAFU-Cattle Protocol B	YOLOv8-Pose	97.13 ± 0.15	74.66 ± 0.47	98.83 ± 0.07	72.36 ± 0.38
NWAFU-Cattle Protocol B	FSMC-Pose	**98.43 ± 0.12**	94.72 ± 0.40	**99.44 ± 0.04**	79.56 ± 0.48
NWAFU-Cattle Protocol B	LEC-Pose	98.31 ± 0.15	**95.81 ± 0.43**	99.39 ± 0.05	**80.03 ± 0.38**

**Table 4:** Source-stratified and near-duplicate-group-disjoint evaluation on NWAFU-Cattle. Bold numerical values indicate the best performance for each accuracy metric under the corresponding protocol.

Method	Train Images	Validation Images	Test Images	Precision	Recall	mAP@50	mAP@50:95
YOLOv8-Pose	1689	248	495	91.84	64.77	91.92	63.28
mtYOLO	1689	248	495	93.12	85.66	94.36	70.48
FSMC-Pose	1689	248	495	**94.27**	87.92	95.11	71.61
LEC-Pose	1689	248	495	93.88	**89.74**	**95.36**	**72.84**

**Table 5:** Computational efficiency of the principal models. Peak training memory uses batch size 16.

Dataset	Method	Parameters (M)	FLOPs (G)	FPS	Peak Inference Memory (GB)	Peak Training Memory (GB)
CattleEyeView	YOLOv8-Pose	3.29	9.23	138.2	1.38	8.62
CattleEyeView	mtYOLO	7.86	18.54	84.6	2.31	12.41
CattleEyeView	YOLOX-Pose (Livestock)	5.72	12.18	119.7	1.76	10.07
CattleEyeView	FSMC-Pose	6.93	15.68	96.8	2.08	11.36
CattleEyeView	LEC-Pose	4.87	12.63	111.6	1.96	10.52
NWAFU-Cattle	YOLOv8-Pose	3.29	9.23	166.4	1.37	8.51
NWAFU-Cattle	mtYOLO	7.86	18.54	105.9	2.22	12.09
NWAFU-Cattle	YOLOX-Pose (Livestock)	5.72	12.18	143.2	1.72	9.86
NWAFU-Cattle	FSMC-Pose	6.93	15.68	117.6	2.01	11.08
NWAFU-Cattle	LEC-Pose	4.87	12.63	132.7	1.78	10.19

**Table 6:** Detailed module-combination ablation on CattleEyeView. “Y” indicates that the component is enabled. Bold numerical values indicate the best performance for each accuracy metric under the corresponding protocol.

ID	ROI	Std. Conv	DCN	Attn.	HM	Off.	Desc.	Con.	Precision	Recall	mAP@50	mAP@50:95	FLOPs	FPS
A0	–	–	–	–	–	–	–	–	63.80	34.80	56.43	33.80	9.20	138.20
A1	Y	–	–	–	Y	–	–	–	64.81	41.87	58.61	34.42	10.70	126.80
A2	Y	–	–	–	Y	Y	–	–	65.36	44.28	59.76	35.14	10.90	123.90
A3	Y	Y	–	–	Y	Y	–	–	65.92	47.63	60.83	35.58	11.50	119.40
A4	Y	–	Y	–	Y	Y	–	–	66.77	50.41	61.92	36.16	11.90	116.70
A5	Y	–	–	Y	Y	Y	–	–	66.51	51.02	62.14	35.98	11.20	121.30
A6	Y	Y	–	Y	Y	Y	–	–	67.34	53.88	62.73	36.43	11.80	116.20
A7	Y	–	Y	Y	Y	Y	–	–	68.13	55.92	63.29	36.78	12.60	112.60
A8	Y	–	Y	Y	Y	Y	Y	–	69.05	60.14	65.17	37.81	12.60	111.90
A9	Y	–	Y	Y	Y	Y	Y	Y	**71.26**	**66.73**	**68.17**	**39.58**	12.60	112.10

**Table 7:** Cosine-similarity analysis of the global ROI descriptor on CattleEyeView.

Pair Category	Pairs	Mean Cosine Similarity	Standard Deviation
Same identity, similar pose	9327	0.861	0.076
Same identity, different pose	8894	0.704	0.118
Different identity, similar pose	9161	0.648	0.105
Different identity, different pose	9478	0.439	0.149

**Table 8:** Cross-backbone validation with HRNet on NWAFU-Cattle [39] under Protocol A (50%/50%). FLOPs include both the shared cattle detector and the HRNet-based pose estimator.

Setting	Precision (%)	Recall (%)	mAP@50:95 (%)	FLOPs (G)	FPS
HRNet baseline	90.43	87.19	63.52	25.2	88
+ ROI	91.58	88.03	64.41	26.4	82
+ ROI + RNet	92.46	89.27	65.83	29.0	78
+ ROI + RNet + PE (w/o Con)	93.21	90.12	66.95	29.0	80
+ ROI + RNet + PE + Con	93.87	90.76	67.94	29.0	79

**Table 9:** Cross-backbone validation with HRNet on NWAFU-Cattle [39] under Protocol B (80%/20%). FLOPs include both the shared cattle detector and the HRNet-based pose estimator.

Setting	Precision (%)	Recall (%)	mAP@50:95 (%)	FLOPs (G)	FPS
HRNet baseline	93.20	90.14	65.27	25.2	88
+ ROI	94.01	90.68	66.42	26.4	83
+ ROI + RNet	94.67	91.37	67.83	29.0	79
+ ROI + RNet + PE (w/o Con)	95.02	92.01	68.94	29.0	80
+ ROI + RNet + PE + Con	95.36	92.48	69.83	29.0	79

**Table 10:** Sample statistics for the CattleEyeView visibility groups. Images and cattle instances may contribute to more than one row when they contain keypoints from multiple visibility groups.

Visibility Group	Images with Group	Instances with Group	Evaluated Keypoints	Proportion (%)
Visible	7669	36,182	604,783	68.26
Partially Occluded	5913	22,749	191,246	21.59
Heavily Occluded	3207	10,613	89,907	10.15
Total valid keypoints	7846	36,914	885,936	100.00

**Table 11:** Occlusion robustness under keypoint visibility grouping on CattleEyeView. Only keypoints in the corresponding visibility group are used for OKS computation.

	Visible			Partially Occluded			Heavily Occluded		
Method	Precision (%)	Recall (%)	mAP@50:95 (%)	Precision (%)	Recall (%)	mAP@50:95 (%)	Precision (%)	Recall (%)	mAP@50:95 (%)
HRNet	53.80	70.42	17.68	43.26	60.73	12.14	31.85	48.68	6.92
YOLOv8-Pose	70.65	43.72	38.91	62.18	34.52	33.24	50.42	24.91	25.73
LEC-Pose	76.45	72.36	44.67	71.18	67.84	40.53	64.28	58.69	35.91

**Table 12:** Occlusion robustness under cattle crowding-level grouping on CattleEyeView. Images are grouped by the number of annotated cattle instances.

	Single Cow			2–3 Cows			More Than 3 Cows		
Method	Precision (%)	Recall (%)	mAP@50:95 (%)	Precision (%)	Recall (%)	mAP@50:95 (%)	Precision (%)	Recall (%)	mAP@50:95 (%)
HRNet	51.62	66.84	15.48	46.10	61.85	12.38	39.42	55.30	8.64
YOLOv8-Pose	68.37	39.86	36.62	63.94	35.28	33.92	57.65	28.47	29.74
LEC-Pose	74.02	70.48	42.35	71.45	67.02	39.83	68.36	61.15	36.92

**Table 13:** Occlusion robustness on the NWAFU-Cattle Protocol B test set.

Visibility Group	Images	Instances	Keypoints	YOLOv8-Pose		mtYOLO		FSMC-Pose		LEC-Pose	
				mAP@50	mAP@50:95	mAP@50	mAP@50:95	mAP@50	mAP@50:95	mAP@50	mAP@50:95
Visible	473	605	7203	98.26	76.88	98.71	79.12	99.34	81.02	99.28	81.47
Partially Occluded	331	412	2164	92.14	63.27	94.06	67.41	95.12	69.18	96.03	72.26
Heavily Occluded	178	221	1051	79.44	47.92	82.67	52.36	84.23	55.18	87.11	59.72

**Table 14:** Profile of publicly available animal pose datasets for supervised target-domain evaluation, grouped by viewpoint, species scope, and scene domain.

Dataset	Viewpoint	Species Scope	Scene Domain
CowPose [70]	Side-view	Cattle	Farm scene
Roboflow-CowPose [71]	Side-view	Cattle	Farm scene
MOUNT-Cattle [62]	Side-view	Cattle	Mounting interaction
DairyGoatMVT [72]	Side-view	Dairy goat	Farm scene
Horse-10 [73]	Multi-view	Horse	Equine scene
BamaPig2D [74]	Multi-view	Pig	Pig motion-capture scene
StanfordExtra [75]	Multi-view	Dog	In-the-wild scene

**Table 15:** Exact image-level split sizes used in the supervised target-domain evaluation. The 1000 labeled training instances are sampled without replacement from the corresponding training partition.

Dataset	Total Images	Training Images	Validation Images	Test Images	Sampled Training Instances
CowPose	2000	1400	200	400	1000
Roboflow-CowPose	1771	1458	209	104	1000
MOUNT-Cattle	4284	3427	428	429	1000
DairyGoatMVT	2108	1476	211	421	1000
Horse-10	8114	5680	811	1623	1000
BamaPig2D	3340	2338	334	668	1000
StanfordExtra	12,000	8400	1200	2400	1000

**Table 16:** Supervised target-domain evaluation on external animal pose datasets. The table reports mAP@50:95 for each named method under the corresponding target-dataset protocol.

Target Dataset	HRNet	YOLOv8-Pose	LEC-Pose
CowPose [70]	65.87	70.21	76.46
Roboflow-CowPose [71]	63.45	67.27	73.62
MOUNT-Cattle [62]	87.27	86.05	89.81
DairyGoatMVT [72]	84.85	82.25	88.42
Horse-10 [73]	58.81	60.00	66.74
BamaPig2D [74]	63.33	60.75	67.28
StanfordExtra [75]	56.83	54.56	63.38

**Table 17:** End-to-end zero-shot cross-dataset evaluation. All models are trained and selected using NWAFU-Cattle, and the target datasets are used only for final testing.

Target Dataset	Method	Common Keypoints	Precision	Recall	mAP@50	mAP@50:95
CowPose	YOLOv8-Pose	12	61.74	47.19	54.83	27.14
CowPose	mtYOLO	12	64.28	50.82	57.26	29.08
CowPose	LEC-Pose	12	66.91	54.37	59.41	31.76
Roboflow-CowPose	YOLOv8-Pose	12	56.48	42.73	49.27	23.61
Roboflow-CowPose	mtYOLO	12	59.73	46.11	52.84	25.96
Roboflow-CowPose	LEC-Pose	12	62.15	49.86	55.16	28.43
MOUNT-Cattle	YOLOv8-Pose	14	53.91	39.64	46.72	21.85
MOUNT-Cattle	mtYOLO	14	59.82	45.36	53.48	25.19
MOUNT-Cattle	LEC-Pose	14	58.77	48.91	52.91	27.08

## Data Availability

The public image datasets are available from the cited sources. To support reproducibility, the fixed NWAFU-Cattle Protocol A and Protocol B image lists, the source-stratified and near-duplicate-group-disjoint split lists, the visibility-group annotations, the external-dataset sampling lists, and the zero-shot keypoint-mapping configuration will be released with the final article and are available from the corresponding authors during review.
